# Differences in the behavior and diet between shoaling and solitary surgeonfish (*Acanthurus triostegus*)

**DOI:** 10.1002/ece3.9686

**Published:** 2023-01-06

**Authors:** Ana Sofia Guerra, Jacey C. Van Wert, Alison J. Haupt, Douglas J. McCauley, Erika J. Eliason, Hillary S. Young, David Lecchini, Timothy D. White, Jennifer E. Caselle

**Affiliations:** ^1^ Department of Ecology, Evolution, and Marine Biology University of California Santa Barbara Santa Barbara California USA; ^2^ Department of Marine Science California State University Monterey Bay Seaside California USA; ^3^ Marine Science Institute University of California Santa Barbara Santa Barbara California USA; ^4^ EPHE‐UPVD‐CNRS PSL University Mo'orea French Polynesia; ^5^ Laboratoire d'Excellence "CORAIL" Paris France; ^6^ Hopkins Marine Station Stanford University Pacific Grove California USA

**Keywords:** coral reef ecology, ecological function, herbivorous fish, intraspecific behavior, schooling, social behavior

## Abstract

Variation in behavior within marine and terrestrial species can influence the functioning of the ecosystems they inhabit. However, the contribution of social behavior to ecosystem function remains underexplored. Many coral reef fish species provide potentially insightful models for exploring how social behavior shapes ecological function because they exhibit radical intraspecific variation in sociality within a shared habitat. Here, we provide an empirical exploration on how the ecological function of a shoaling surgeonfish (*Acanthurus triostegus*) may differ from that of solitary conspecifics on two Pacific coral reefs combining insight from behavioral observations, stable isotope analysis, and macronutrient analysis of gut and fecal matter. We detected important differences in how the social mode of *A. triostegus* affected its spatial and feeding ecology, as well as that of other reef fish species. Specifically, we found increased distance traveled and area covered by shoaling fish relative to solitary *A. triostegus*. Additionally, shoaling *A. triostegus* primarily grazed within territories of other herbivorous fish and had piscivorous and nonpiscivorous heterospecific fish associated with the shoal, while solitary *A. triostegus* grazed largely grazed outside of any territories and did not have any such interactions with heterospecific fish. Results from stable isotope analysis show a difference in δ15N isotopes between shoaling and solitary fish, which suggests that these different social modes are persistent. Further, we found a strong interaction between social behavior and site and carbohydrate and protein percentages in the macronutrient analysis, indicating that these differences in sociality are associated with measurable differences in both the feeding ecology and nutrient excretion patterns. Our study suggests that the social behavior of individuals may play an important and underappreciated role in mediating their ecological function.

## Graphical abstract

In this study, we characterized movement and foraging‐associated behaviors of shoaling and solitary surgeonfish on coral reefs. We found differences in movement, grazing, and interactions with heterospecific fish between shoaling and solitary fish. These observations suggest that the social behavior of individuals may play an important role in mediating their ecological function.

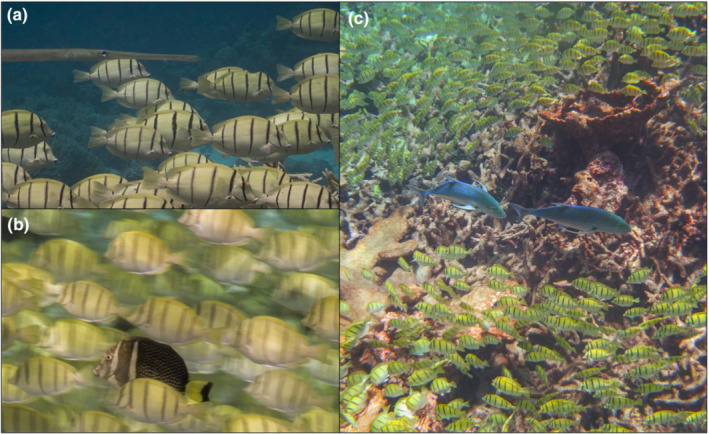

## 1 INTRODUCTION

Shifts and individual variations in animal behavior can influence the functioning of the ecosystems they inhabit. For example, herbivorous animals may shift their foraging habitat to avoid predation, which alters primary production, distribution of their food sources, and nutrient cycling (Dill et al., 2003; Heithaus et al., 2008; Stief & Hölker, 2006). Research on how the “ecology of fear” shapes animal movement and habitat preferences has become increasingly common (Zanette & Clinchy, 2019). Yet, another candidate behavior that has the potential to influence ecosystems is social behavior; i.e., whether members of a species that exhibit intraspecific variation in their sociality tend to live and operate in groups or spend all or most of their time alone. Previous work has shed light on how these differences in sociality may shape ecological outcomes. For example, seed dispersal by harvester ants varies depending on whether the ants are solitary or social foragers; consequently, plant community patterns differ in the foraging grounds of solitary and social ants (Avgar et al., 2008). Further, in the Great Lakes region of North America, when wolves form larger pack sizes, their moose kill rate increases (Post et al., 1999). This increased kill rate then influences moose abundance and cascades to reduced browsing and greater understory growth (Post et al., 1999).

As a taxon that inhabits many diverse and important marine and freshwater ecosystems, fish ecological function has received significant attention (Mumby et al., 2006; Nash et al., 2013). On coral reefs, considerable effort has been dedicated to understanding the ecological role of heavily fished herbivorous species such as parrotfish (Labridae) and surgeonfish (Acanthuridae), that can create suitable habitat for coral recruitment and mediate coral‐macroalgae interactions through their grazing behavior (Hughes et al., 2007; Mumby et al., 2006) and provide nutrients through excretion (Allgeier et al., 2017; Burkepile et al., 2013). Many of these herbivorous coral reef fish species (e.g., *Acanthurus, Scarus*, and *Chlorurus* sp.) exhibit intraspecific variation in social behavior, with certain individuals in the same area forming shoals, a group of fish swimming together in a loose or organized fashion, while others operate alone or in very small aggregations. This coupled with their often outsized functional importance makes coral reef fishes highly suitable subjects for examining how differences in social behaviors affect ecological outcomes.

The majority of research on shoaling behavior has focused on the evolutionary tradeoffs of shoaling, mechanics and hydrodynamics, and predator avoidance (Krause & Ruxton, 2002; Pitcher, 1986); however, less is known about the ecological function of shoaling behavior. Previous work has provided preliminary insight into some of these linkages between shoaling and ecology. For example, on Caribbean reefs, solitary blue tang (*Acanthurus coeruleus*) primarily graze in undefended areas while shoaling blue tang often invade and graze down other herbivorous fishes' territories (Foster, 1985; Robertson et al., 1976). Additionally, parrotfish have been found to graze algae at faster rates when in shoals, creating a more suitable habitat for coral growth (Welsh & Bellwood, 2012b). Further, shoals of grunts that shelter around coral heads are important for creating nutrient hotspots of bioavailable nitrogen that can foster coral growth (Meyer et al., 1983; Shantz et al., 2015). Recent evidence suggests that some grouping behavior in fish, such as shoaling, could be vulnerable to change in a heavily fished ocean (Guerra et al., 2020; Sbragaglia et al., 2021); thus, heightening the importance of understanding how social behavior shapes ecosystems.

Here, we examine how the ecological function of shoaling surgeonfish may differ from that of solitary conspecifics in the field on n two different tropical Pacific reefs. We compared foraging, movement, and interspecific interactions of shoaling and solitary convict surgeonfish (*Acanthurus triostegus*), an abundant herbivore that has a variable tendency to form large shoals (1000 fish), small‐ and medium‐sized shoals that range from 5–500 individuals, or forage alone (Barlow, 1974; Randall, 1961; pers. obs.). Using behavioral focal follows, we recorded information on four parameters: (1) distance traveled, (2) area covered, (3) grazing invasions of fish territories, and (4) associations by heterospecific fish. From collected specimens of shoaling and solitary fish, we also measured data on two parameters: (1) stable isotope values of muscle tissue and (2) macronutrient quantities in stomach and fecal contents. Together, this suite of measures provided strong evidence that sociality in this species controls important ecological outcomes.

## 2 METHODS

### 2.1 Study sites and species

The study was conducted on the Pacific coral reefs of Palmyra Atoll (5°53′N, 162°5′W) and Mo'orea Island (17°32′S 149°50′W). Palmyra Atoll (USA) is a remote uninhabited island that forms part of the northern Line Islands archipelago in the Central Pacific. Mo'orea (French Polynesia) is an inhabited island that forms part of the Society Islands archipelago in the South Pacific.

*Acanthurus triostegus* are common throughout coral reefs in the tropical Indo‐Pacific Ocean and it is a grazing reef herbivore that feeds primarily on filamentous algae and cylindrical algae, as well as some cyanobacteria, foliose algae, and calcareous algae (Nalley et al., 2021; Randall, 1961). Metabarcoding of diet contents of *A. triostegus* in Hawai'i identified 64 unique diet items, with Rhodophyta dominating the abundance, followed by ochrophyta and cyanobacteria (Kelly et al., 2016; Nalley et al., 2021). These abundant coral reef fish exhibit both shoaling and solitary behavior on both islands (Guerra et al., 2022), providing an excellent opportunity to explore the ecology of shoaling behavior. Surveys of benthic habitats of backreef habitats of Palmyra Atoll and Mo'orea Island are summarized in Appendix B and Figure A2.

**FIGURE A1:**
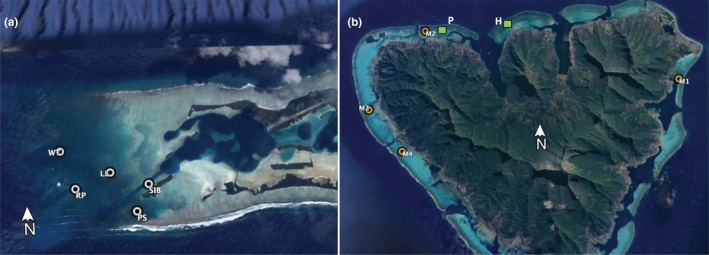
Field sites on (A) Palmyra Atoll, USA and (B) Mo'orea, French Polynesia. Circles represent behavioral observation sites, and squares (sites P and H, only on Mo'orea (B), represent sampling sites). GPS Coordinates for sites can be found in data repository at https://doi.org/10.25349/D94617.

**FIGURE A2:**
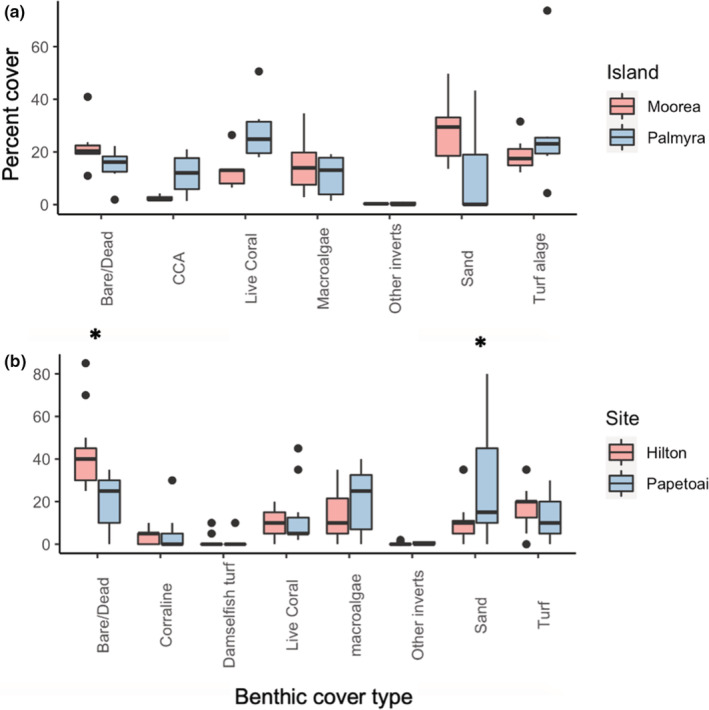
Benthic cover surveys (described in Appendix C) categories revealed (A) no significant differences within any cover type either between Mo'orea Island and Palmyra Atoll but (B) a lower amount of bare rock/dead coral and higher amount of sand in Papetoai between focal sites within Mo'orea Island (significant differences marked with an asterisk).

Fish behavioral follows were conducted at four sites on the backreef of Palmyra Atoll and four sites on the backreefs of Mo'orea during July and August of 2017 on Palmyra Atoll and 2018 on Mo'orea, and fish collections were conducted at two backreefs sites in November of 2018 on Mo'orea (Figure A1).

Although it is not clear how fixed the associations are between solitary and shoaling life modes within individuals, our preliminary data suggests that these behavior modes may remain fixed for at least moderate durations. For a small number of focal individuals, we combined photo records using diagnostic natural variation in *A. triostegus* coloration (Figure A4 and A5) to show fidelity to either small (i.e., ≤3 individuals) groups (*n* = 5 individual tracked fish) or too large (i.e., >50 individuals) groups (*n* = 7 individual tracked fish) over the entirety of a 20‐day observation period (Appendix C). These patterns hold for longer time periods and over a year later we resighted fish exhibiting consistent social behavior as before: two solitary individuals (21 and 36 months later) and two shoaling individuals (21 months later; Appendix C).

### 2.2 Behavioral observations

We conducted 30–60 min focally follows on both islands to measure foraging, distance traveled, 95% KUD (kernel utilization density), and interspecific interactions by shoaling and solitary *A. triostegus*. Snorkeling observers (four observers on Palmyra Atoll, two on Mo'orea, lead observer ASG was present at both islands to ensure methodological consistency and observer training) followed solitary or shoaling *A. triostegus* while towing a GPS device that recorded location every 60 s. Initial follows were conducted at both islands to assess the appropriate distance for following fish that would not impact normal foraging nor initiate a flight response, which we defined as moving away from the observer at an accelerating speed, or quickly changing swimming directions (Gotanda et al., 2009). Every 60 s, the observer noted shoal size (if applicable), presence or absence of grazing behavior, whether a grazing event constituted a territorial invasion, and associations with heterospecific fish species (Table 1, Table A1 for species list). Interspecific interactions that occurred during each observation minute were recorded and described as either: “territorial invasions,” where grazing behavior by *A. triostegus* elicited territorial defense behavior from heterospecific fish (i.e., territories not mapped based on benthic visual cues but rather demarked based on observed territory defense behaviors; Dromard et al., 2013; Foster, 1985), “nonpredatory association,” where herbivorous heterospecific fish associated with the focal school or fish (Alevizon, 1976), or “predatory association,” where the interaction involved a piscivore or invertivore (Madin & Madin, 2011; Ormond, 2009; Table 1). Predatory and nonpredatory associations were defined as a fish of a different species moving in the same direction and in close proximity to *A. triostegus* for five or more consecutive minutes. Observations on shoals were done by recording behavioral information based on the behavior of 50% or more of the individuals in the shoal (e.g., shoal was recorded as “grazing” if at least half of the shoal was in a nose‐down grazing position at the 60 s mark). If a shoal was widely dispersed or in a line formation, the observer followed the last 1/3 of the shoal and recorded the information for that subset of the shoal. If an observer lost sight of a solitary fish or shoal of fish, they searched for the fish for up to 2 min. If after 2 min the fish were not located, the focal follow was terminated.

**TABLE 1:** Experimental system framework for observations of solitary and shoaling behavior of *Acanthurus triostegus*

Observation	Description
Distance traveled	Total linear distance traveled (standardized per minute of follow)
Area covered (95% KUD)	Area covered in 25‐min follow
Grazing	Proportion of follow spent in nose‐down grazing position (measured every 60 s)
Territorial invasions	Proportion of grazing events that were territory invasions
Nonpredatory fish associations	Proportion of time heterospecific nonpredatory fish was associated
Predatory fish associations	Proportion of time heterospecific predatory fish was associated

We found a significant difference in time spent in a grazing position and distance traveled in the first 5 min of observation, relative to subsequent five‐minute bins, suggesting the presence of an observer effect; therefore, we removed the first 5 min of every follow. As fish observations had different durations (30–60 min), distance traveled was standardized per minute (divided over total follow duration), and analysis of KUD was done by capping all follows at 30 min (total of 25 min excluding initial 5 min), as total follow time may affect total space use. The proportion of time spent in grazing position, proportion of territorial invasions out of all grazing events, and associations by heterospecific fish (predatory and nonpredatory) were calculated across all follow durations. We computed distance traveled using the *adehabitatLT* package in R and 95% utilization kernel using a biased random bridge method in the *adehabitatHR* package in R (version 4.0.3) (Calenge, 2006; R Core Team, 2020; RStudio Team, 2020).

### 2.3 Analysis

All computations were conducted in R (version 4.0.3) using R studio and the *tidyverse* package (RStudio Team, 2020; Wickham et al., 2019). We used linear mixed‐effects models to explain variations in distance traveled, 95% KUD, proportion of grazing events that were territorial invasions, and associations with predatory and nonpredatory fish for *A. triostegus* on Palmyra Atoll and Mo'orea. We specified full models with the *nlme* package (Pinheiro et al., 2022), using distance traveled (per min), 25‐min 95% KUD, proportion of grazing events that were territorial invasions, associations with predatory fish, and associations with nonpredatory fish as response variables; social status (shoaling or solitary) as a fixed effect; and site, island, and time of day as random effects, since time of day can affect surgeonfish behavior (Table A2; Montgomery et al., 1989, Zemke‐White et al., 2002). As our behavioral observations were done on shoals of different sizes, we used linear mixed‐effects models fit by maximum likelihood (ML) to explain variations in the response variables mentioned above for shoaling *A. triostegus* only. We specified full models the same as above using the *nlme* package and added shoal size as a fixed effect instead of social status (shoaling or solitary). Best‐fit models were selected according to small‐samples corrected AIC (AICc) using the package *MuMIn* (Barton, 2020). For 95% KUD, the data distribution was non‐normal; thus, we transformed the data with a log normal transformation as suggested by Zuur et al. (2009). As time spent in grazing position data was collected differently between shoaling and solitary fish (shoal‐scale vs. individual), we fit models to compare this metric between shoaling and solitary fish.

### 2.4 Fish sampling

To directly test whether any differences in foraging and movement behavior that were detected between shoaling and solitary *A. triostegus* affected their diet and trophic ecology, we collected 100 individuals (25 shoaling and 25 solitary from two different sites in Mo'orea only; Figure A1, sites P and H, which are ~3 km apart and separated by a channel) to compare muscle tissue stable isotope values and assess the nutritional quality of stomach contents and fecal matter. Fish were collected using hand spears.

Shoals of 50 individuals or larger were defined as ‘shoaling fish’ for this study. All fish were collected between 1000–1600 h, to ensure the fish had been feeding for sufficient time to have contents in their stomach (i.e., based on gut throughput time data from congeners in Polunin et al., 1995). Following collection, fish were kept on ice for a maximum of three hours before processing. During processing, we recorded body morphometrics (standard length, wet weight), sampled muscle tissue for stable isotope analysis, removed and weighed the gut, and stomach contents and feces (determined as contents in terminal 1 cm of intestine) were dissected and stored separately in a −20°C freezer for each fish.

### 2.5 Stable isotope analysis

We conducted stable isotope analysis of δ^13^C and δ^15^N isotope ratios to explore potential foraging differences between shoaling and solitary *A. triostegus*. Stable isotopes from certain tissues can be useful indicators of diet over longer time periods than those available from stomach content analysis (Matley et al., 2016). Analysis of isotopic signatures can determine differences in dietary and trophic niche between coral reef fish species and individuals within a species (Eurich et al., 2019). We used isotopic signatures from muscle tissue to infer *A. triostegus* diet, as the integration rate for fish muscle tissue is found to be reliable over long periods of time (Matley et al., 2016). Prior to isotopic analysis, muscle tissue was lyophilized for 48 h, homogenized, and ~1.3 mg were loaded into tin capsules, which were sent to the University of California, Davis Stable Isotope Facility for analysis. Samples were analyzed for δ^13^C and δ^15^N isotopes using a PDZ Europa ANCA‐GSL elemental analyzer interfaced with a PDZ Europa 20–20 isotope ratio mass spectrometer (Sercon Ltd.).

We used linear models to explain variations in nitrogen and carbon stable isotope values for shoaling and solitary *A. triostegus* on Mo'orea. We specified full models using the *nlme* package (Pinheiro et al., 2022), with δ^15^N and δ^13^C as response variables; and social status (shoaling or solitary), site, and fish size (standard length) as predictors (Table A2). Best‐fit models were selected according to small‐samples corrected AIC (AICc) using the package *MuMIn* (Barton, 2020), which compares all possible iterations of combined and individual predictors from the full model. Additionally, we generated Bayesian standard ellipses (40% confidence level) for each social behavior (shoaling or solitary) and backreef site using the SIBER package to estimate isotopic niche space (Jackson et al., 2011). We compared the size of the ellipses by fitting Bayesian models adjusted for small sample sizes (SEAc) and calculated overlap in ellipse area between the two sites and social behaviors, which can be used to determine overlap in diets and niche space (Eurich et al., 2019). Shared overlap of >60% was considered a significant shared niche space (Eurich et al., 2019; Schoener, 1968).

### 2.6 Macronutrient analysis

We selected a subset of 39 fish based on the results of the stable isotope analysis for analyzing stomach contents and fecal matter macronutrients. As δ^15^N values for shoaling and solitary fish were significantly different (as discussed below), we elected to analyze the stomach contents of 19 of the shoaling fish (9 from site P and 10 from site H) with the lowest δ^15^N values and 20 of the solitary fish with the highest δ^15^N values (10 from each site). By selecting these most isotopically divergent individuals, we aimed to characterize with greater clarity any macronutrient differences in diet and fecal content that may occur between these behavior modes.

Stomach contents and feces were analyzed for moisture, protein, carbohydrate, lipid, and ash content to the nearest 0.00001 g (Mettler Toledo MS105DU). We first freeze‐dried samples in a lyophilizer for 36 h to remove and measure water content. We then manually homogenized each sample with a conical glass homogenizing pestle and measured 10 mg of sample into homogenizing 2 ml screw cap vials for further homogenization for protein analysis. We diluted these aliquots with milliQ water with a dilution factor of 100 and homogenized the samples using 10 mg 0.5 zirconium oxide beads at 6 m s^−1^ for four 30 s cycles (Fisher Brand Bead Mill 24). These homogenized aliquots and the remainder of the sample were stored at −20°C until further use. To measure total protein, we thawed the homogenate and precipitated the protein from the sample with bovine albumin serum (BSA) standard and 72% trichloroacetic acid (TCA), removed the supernatant, and then followed a microplate BCA assay protocol (Thermoscientific Pierce BCA Kit) and measured absorbance at 562 nm in triplicate (Mann & Gallager, 1985). We used standard curves with *R* ^2^ > 0.98. For lipids, we followed a modified micro version of the Folch method (Folch et al., 1957; Johnson et al., 2017; Mann & Gallager, 1985). Briefly, we measured 5–20 mg of sample into solvent‐washed test tubes in duplicate, added 100 ul water and 1.5 ml chloroform:methanol (1:2), incubated at 4°C for 10 min, and centrifuged (4000 rpm, 5 min). We removed the supernatant and re‐extracted the remaining sample with 1.5 ml chloroform: methanol (2:1) and pooled the supernatants. Finally, we added 950 μl NaCl (0.7%), incubated the mixture at 4°C for 30 min, centrifuged, quantified the volume in the lower phase, and added 1 ml of the lower phase to a preweighed aluminum weigh boat. We dried the sample overnight, reweighed the remaining lipid, and extrapolated the entire bottom layer volume for lipid content. To measure ash content, we precombusted aluminum weigh boats at 450°C for 6 h and preheated the samples in an oven at 100°C overnight to ensure full water loss. We then combusted preweighed samples in a muffle furnace for 6 h at 450°C and reweighed samples to measure ash content. Finally, we estimated total carbohydrates using a method commonly used for estimating carbohydrate content in food, as carbohydrates = 100–proteins–lipids–ash, where variables are in % dry weight (Opstvedt et al., 2003; Rempel et al., 2022; Southgate, 1969).

We used linear models to explain variations in macronutrients for *A. triostegus* on Mo'orea. We specified full models using the *nlme* package (Pinheiro et al., 2022), using percent dry matter of protein, carbohydrates, and lipids in stomach contents and feces as response variables; social status (shoaling or solitary), site, the interaction of social behavior and site, and fish size (standard length) as predictors (Table A2). Best‐fit models were selected according to small‐samples corrected AIC (AICc) using the package *MuMIn* (Barton, 2020), which compares all possible iterations of combined and individual predictors from the full model.

## 3 RESULTS

### 3.1 Behavioral observations

We conducted a total of 94 behavioral follows across both islands; 17 solitary and 19 shoaling fish follows on Palmyra Atoll, and 37 solitary and 21 shoaling fish follows on Mo'orea. All follows were at least 25 min in duration and the majority (69) were 55 min in duration. Observations of shoaling fish were distributed across shoal sizes of 25–500 fish.

**FIGURE 1:**
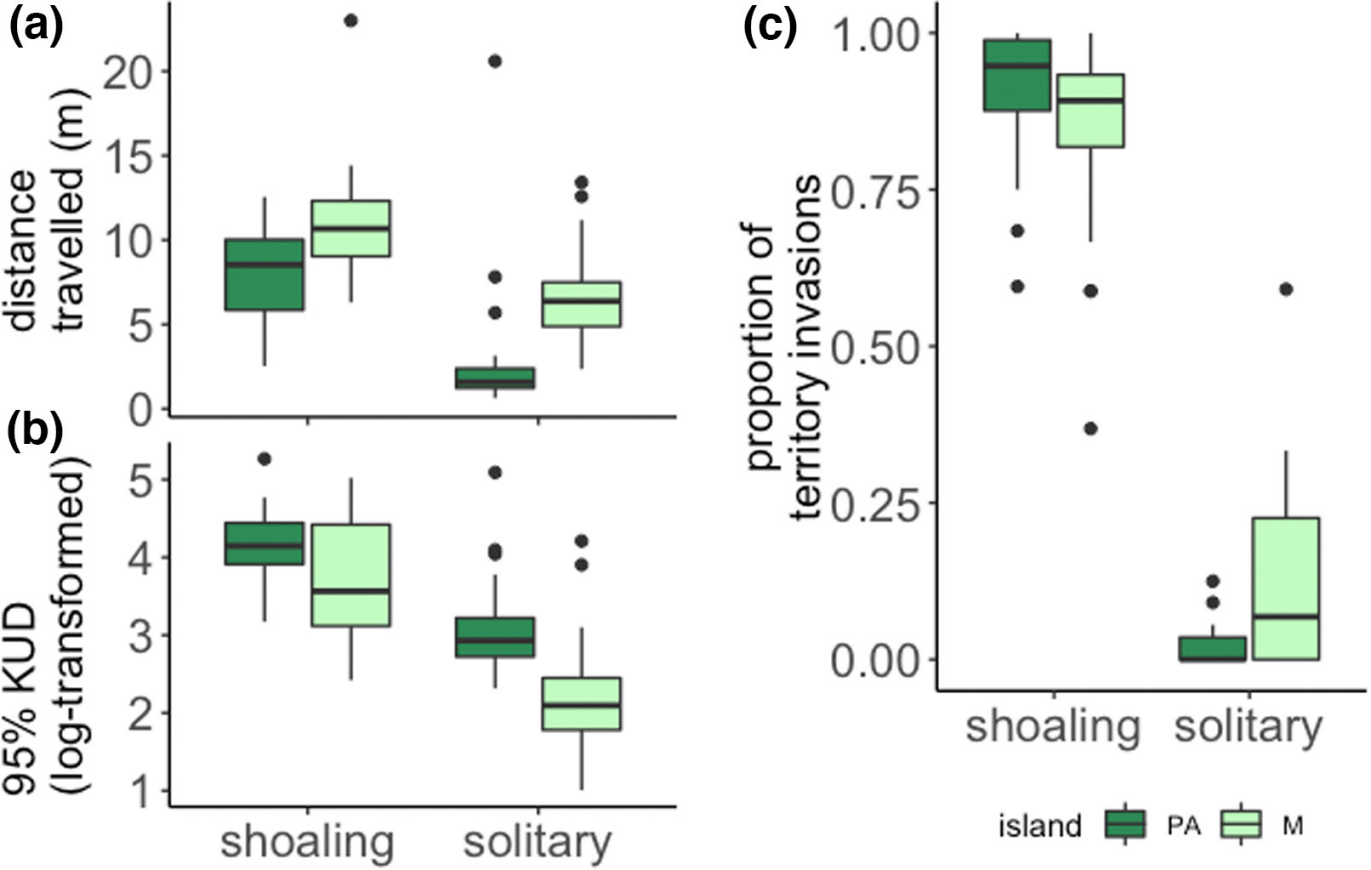
(a) Distance traveled (measured in meters and standardized by minutes of observation), (b) 25‐min 95% kernel utilization distribution (KUD), and (c) proportion of grazing events that were territory invasions for solitary and shoaling *Acanthurus triostegus* on Palmyra Atoll (PA) and Mo'orea (M)

Distance traveled (in meters, standardized by observation minute) is best predicted by a model that includes social behavior (shoaling vs. solitary) as a fixed effect and predicts that solitary *A. triostegus* travel 4.5 m less per minute than shoaling fish (Figure 1a, Table 2). The best‐fit model for predicting distance traveled by shoals did not include shoal size (Table A3). Similar to results for distance traveled, the 95% KUD for 25‐min follows is best predicted by a model that includes social behavior (shoaling or solitary) as a fixed effect and suggests solitary fish cover less area than shoaling fish (Figure 1b, Table 2). The best‐fit model for predicting 95% KUD by shoals did not include any fixed effects, but the next best‐fit model included shoal size as a fixed effect (Table A3).

**TABLE 2:** Best‐fit linear mixed models for explaining variations in distance traveled and 95% KUD for shoaling and solitary *Acanthurus triostegus*

Fixed effect	Distance traveled^a^				95% KUD^a^			
	Estimate	SE	*t*‐value	*p*‐value*	Estimate	SE	*t*‐value	*p*‐value*
Intercept	9.46	1.66	5.69	<.005	3.96	0.30	13.37	<.005
Social behavior (solitary)	−4.45	0.72	−6.18	<.005	−1.25	0.14	−8.88	<.005
**Random effect**	**Variance**	**SD**			**Variance**	**SD**		
Time of day	0.00	0.00			0.00	0.00		
Island	4.97	2.23			0.14	0.38		
Site (within island)	0.00	0.00			0.06	0.25		

^a^ Distance traveled *R* ^2^ = 0.46, 95% KUD *R* ^2^ = 0.60.

* *p*‐value is calculated using the Wald chi‐square test.

Mean proportion of time *A. triostegus* spent in a grazing position during an observational follow was 0.58 (PA)‐0.62 (M) in a shoal and 0.51(PA)‐0.60 (M) while solitary (Table A4). The proportion of grazing events that were invasions of territory was 0.90 ± 0.12 (SD) for shoals on Palmyra Atoll and 0.83 ± 0.16 for shoals on Mo'orea (Figure 1c, Table A4). For solitary fish, territorial invasions comprised only 0.02 ± 0.04 and 0.13 ± 0.14 of grazing events on Palmyra Atoll and Mo'orea, respectively (Table A4). The species whose territories were most commonly invaded were *Stegastes nigricans*, *Acanthurus lineatus*, *Acanthurus nigricans*, and *Ctenochaetus striatus* on Palmyra Atoll, and *Stegastes nigricans*, *Zebrasoma scopas*, *Acanthurus nigrofuscus,* and *Ctenochaetus striatus* on Mo'orea. Invasions to *S. nigricans* algal gardens accounted for 0.49 ± 0.37 and 0.30 ± 0.29 of territorial invasions on Palmyra Atoll and Mo'orea, respectively. Proportion of grazing events that were territorial invasions is best predicted by a model that includes social behavior as a fixed effect, with invasions being more prevalent with fish in shoals (Table 3, Figure 1c).

**TABLE 3:** Best‐fit linear mixed models for explaining variations in territorial invasions, predatory fish associations, and nonpredatory fish associations for shoaling and solitary *Acanthurus triostegus*

Fixed effect	Territorial invasions^a^				Nonpredatory fish^a^				Predatory fish^a^			
	Estimate	SE	*t*‐value	*p*‐value*	Estimate	SE	*t*‐value	*p*‐value*	Estimate	SE	*t*‐value	*p*‐value*
Intercept	0.87	0.21	40.52	<.005	0.42	0.06	6.89	<.005	0.27	0.09	2.88	<.01
Social behavior (solitary)	−0.77	0.03	−27.47	<.005	−0.41	0.05	−8.45	<.005	−0.24	0.04	−5.62	<.005
**Random effect**	**Variance**	**SD**			**Variance**	**SD**			**Variance**	**SD**		
Time of day	0	0			0	0			0	0.06		
Island	0	0			0	0.07			0.01	0.11		
Site (within island)	0	0.01			0	0.03			0.01	0.08		

^a^ Territorial invasions *R* ^2^ = 0.89, nonpredatory fish *R* ^2^ = 0.48, predatory fish *R* ^2^ = 0.52.

* *p*‐value is calculated using the Wald chi‐square test.

**FIGURE 2:**
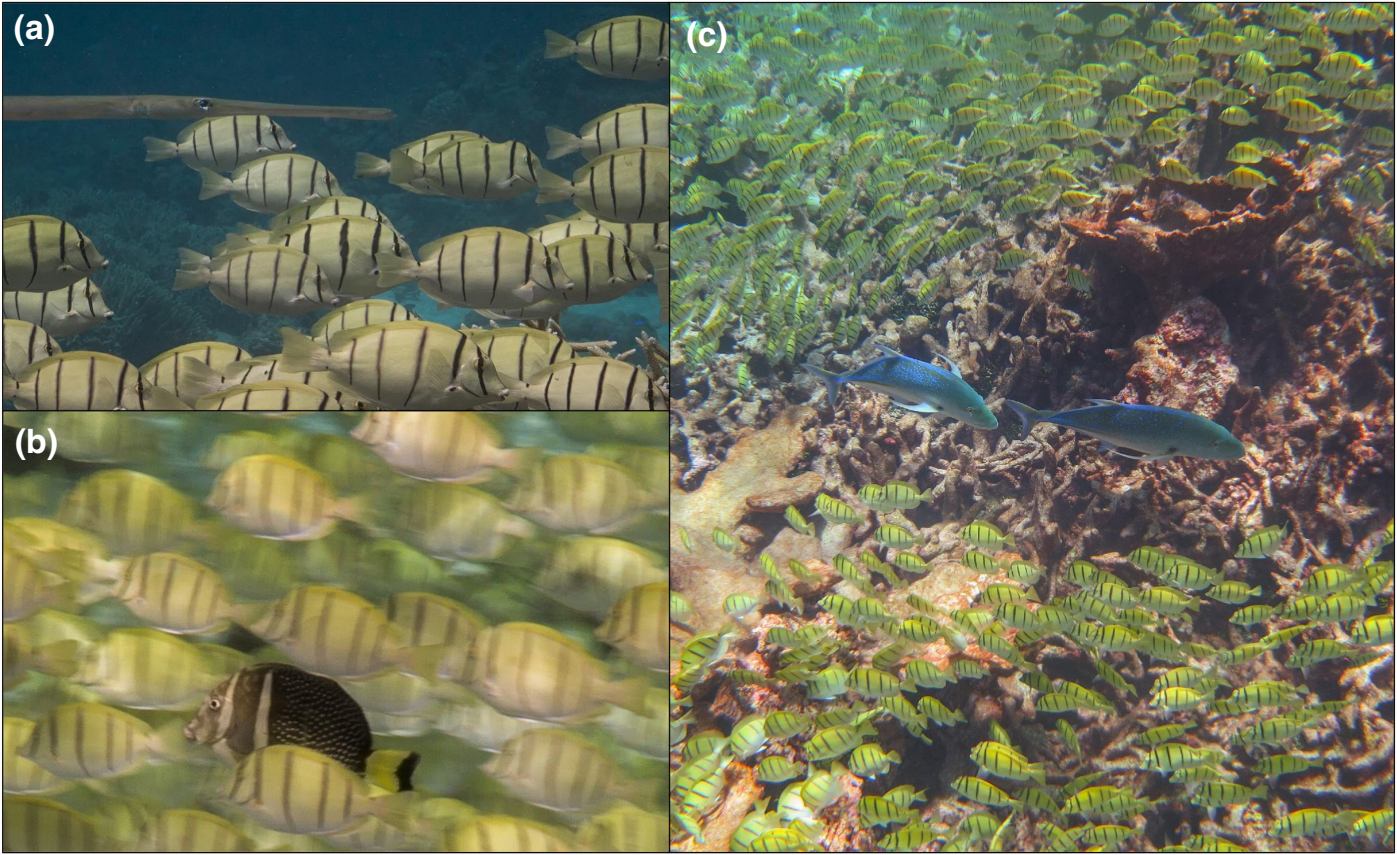
Heterospecific fish associated with *Acanthurus triostegus* shoals: (a) *Fistularia commersonii* (carnivore, not predator of adult *A. triostegus*) on Mo'orea, (b) *Acanthurus guttatus* (herbivore) on Mo'orea, and (c) *Caranx melampygus* (carnivore, can predate *A. triostegus*) on Palmyra Atoll. Photographs by ASG.

Nonpredatory heterospecific fish were associated with 95% (18/19) of all follows of *A. triostegus* shoals on Palmyra Atoll and 71% (15/21) on Mo'orea (Table A4; Figure 2). Species associated were *Acanthurus blochii*, *Acanthurus xanthopterus, Chlorurus spilurus*, *Kyphosus* sp., *Mellycthis niger*, and *Scarus psittacus* on Palmyra Atoll, and *Acanthurus guttatus*, *Cantherhinis dumerilii*, *Chlorurus spilurus*, *Ctenochaetus striatus*, and *Scarus psittacus* on Mo'orea. Nonpredatory fish spent an average of 0.54 ± 0.35 proportion of the follow with shoals on Palmyra Atoll and 0.30 ± 0.32 on Mo'orea. No solitary fish had nonpredatory fish associations on either island. Interspecific associations by nonpredatory fish were best predicted by a model that includes social behavior as a fixed effect, with shoaling behavior increasing the likelihood of the association (Table 3).

Predatory fish were associated with all shoals (19/19) on Palmyra Atoll, and 24% (5/21) on Mo'orea had a predatory fish associated with the shoal at some point during the follow (Figure 2). Primary species associated were *Aulostomus chinensis*, *Carcharhinus melampygus*, *Cephalopholis argus*, *Caranx melampygus*, and *Lutjanus bohar* on Palmyra Atoll, and *Aulostomus chinensis*, *Caranx melampygus*, and *Fistularia commersoni* on Mo'orea. Predatory fish spent an average of 0.47 ± 0.28 proportion of the follow with shoals on Palmyra Atoll and 0.12 ± 0.27 on Mo'orea (Table A4). No solitary fish had nonpredatory fish associations on either island. Interspecific associations by predatory fish were best predicted by a model that includes social behavior as a fixed effect, with shoaling behavior increasing the likelihood of the association (Table 3).

### 3.2 Stable isotope analysis

**FIGURE A3:**
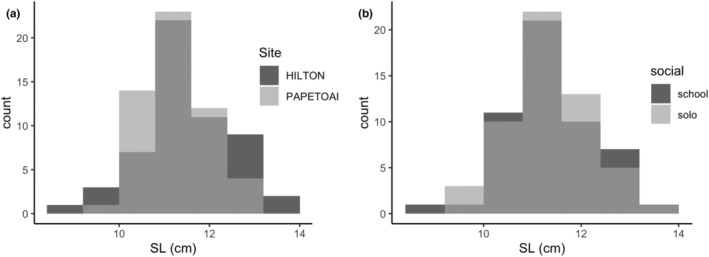
Histograms of sampled fish sizes (standard length in centimeters) across the two sites on Mo'orea, French Polynesia (A), and distribution across the two social modes: shoaling and solitary (B).

**FIGURE A4:**
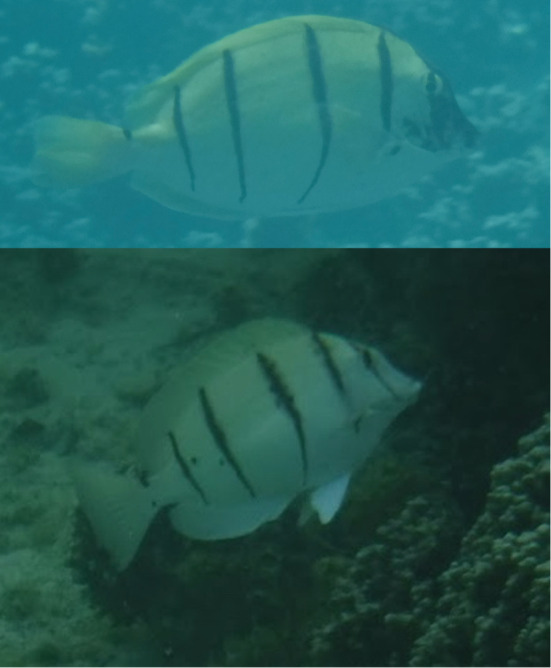
Examples of the distinguishing markings of *Acanthurus triostegus* found on the reefs of Mo'orea. Top image shows a fish with distinct melanistic facial markings and bottom image shows a fish with distinguishing spots on the right flank, distinct spotting along the third bar, and an incomplete bar in the caudal peduncle.

**FIGURE A5:**
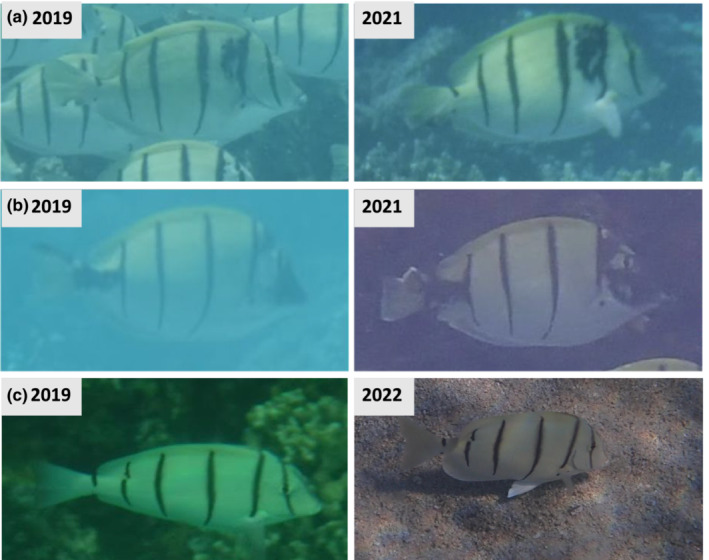
(A and B) Two shoaling *Acanthurus triostegus* sighted in 2019 (first column) and their potential resights in 2021 with growth to melanistic patterns. Fish A shows a fish with a dark mottling that contains a light vacuoule after the second bar, a thick third bar, and a notch on the ventral side of the caudal fin in 2019. In 2021 the caudal fin notch is still present, the third bar appears a bit thicker, and the mottling behind the second bar has grown, but the vacuole is still present. Fish B in 2019 has dark patterns along the nose and in the forehead area between the first and second bar, thin bars along the body, and then a thick stripe of mottling between the 5th and 6th bars, extending out into the caudal peduncle. The dorsal side of the caudal fin also has dark mottling. In 2021 the facial markings appear to have grown to almost fully occupy the anterior side of the second bar. The stripe along the posterior end appears similar, although the caudal fin has been damaged and it is not possible to assess the markings along its dorsal side. (C) A solitary *A. triostegus* sighted in 2019 and potential resight in 2022 with same melanistic patterns. The fish shows an interruption along the dorsal portion of the 5^th^, an incomplete bar in at the caudal peduncle, and otherwise complete bars with no mottling.

We collected 100 *A. triostegus* for stable isotope analysis from two sites on Mo'orea (P and H on the map in Figure A1; 25 shoaling and 25 solitary fish from each site). Shoal sizes ranged up to 500 individuals. Average fish size (standard length) was not significantly different across sites and social behavior (Table A5, Figure A3).

**FIGURE 3:**
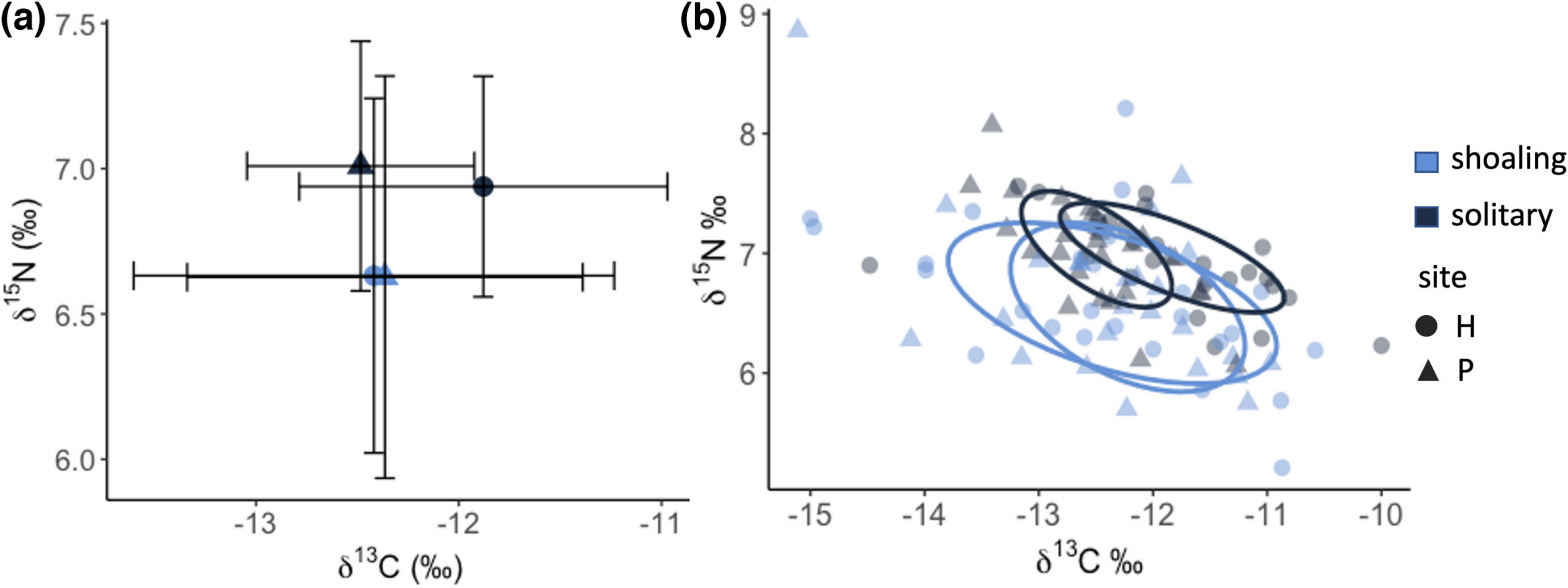
δ^15^N and δ^13^C (‰) signatures of shoaling and solitary *Acanthurus triostegus* tissue samples collected at two sites on Mo'orea. (a) Biplot of isotopic signatures where points are group means, and error bars represent standard deviation. (b) Isotopic area overlap of shoaling and solitary fish samples. Standardized Bayesian ellipse areas (SEAc) are depicted by solid lines, and values for δ^15^N and δ^13^C are expressed in ‰.

The mean value for samples from shoaling fish was δ^13^C: −12.42 ± 1.19 (SD), δ^15^N: 6.63 ± 0.61 (SD) at site H, and δ^13^C: −12.36 ± 0.98 (SD), δ^15^N: 6.63 ± 0.69 (SD) at site P (Figure 3a). For solitary fish, the mean value for samples from site H were δ^13^C: −11.88 ± 0.91, δ^15^N: 6.94 ± 0.38, and δ^13^C: −12.48 ± 0.56,™ ^15^ N: 7.01 ± 0.43 from site P (Figure 3a). δ^15^N is best predicted by a model that includes social behavior and fish size (SL; Table A6); however, this model result was primarily driven by one very small fish (SL < 9 cm) and one very large fish (SL > 13.5 cm) that were outliers in our size distribution (Figure A3). Excluding these two outliers, δ^15^N is best predicted by a model that includes only social behavior (Table 4), with higher δ^15^N values in solitary *A. triostegus*. δ^13^C is best predicted by a model that includes site and fish size, and this best‐fit model is maintained even with the exclusion of the two fish outliers (Table 4; Table A6). In this best‐fit model, δ13C values are lower at site P and decrease with decreasing fish size (Table 4).

**TABLE 4:** Best‐fit linear models for explaining variations in δ^15^N and δ^13^C stable isotope values of muscle tissue of shoaling and solitary *Acanthurus triostegus* ^a^

𝜹^15^N					𝜹^13^C			
Coefficient	Estimate	SE	*t*‐value	*p*‐value	Estimate	SE	*t*‐value	*p*‐value
Intercept	6.64	0.07	90.29	.00	−6.88	1.18	−5.82	.00
Social behavior (solitary)	0.33	0.1	3.23	.002				
SL (cm)					−0.45	0.10	−4.42	.00
Site (P)					−0.41	0.16	−2.6	.01

𝜹^15^N pseudo‐*R* ^2^ = 0.03, 𝜹^13^C pseudo‐*R* ^2^ = 0.13.

^a^ These models do not include two‐size outlier fish.

Samples from shoaling fish at both sites had a higher standard ellipse area (P: 1.85, H: 2.04) than solitary fish (P: 0.57, H: 0.89). Shoaling fish had a significant overlap (77%) in shared isotopic niche space across the two sites (Figure 3b). Solitary fish had a nonsignificant overlap of 27% in isotopic niche space across the two sites (Figure 3b). Overlap in isotopic niche space between shoaling and solitary fish was nonsignificant across the two sites: at site P, shoaling and solitary fish had an overlap in isotopic niche space of 26% and of 16% at site H (Figure 3b).

### 3.3 Macronutrients

**FIGURE 4:**
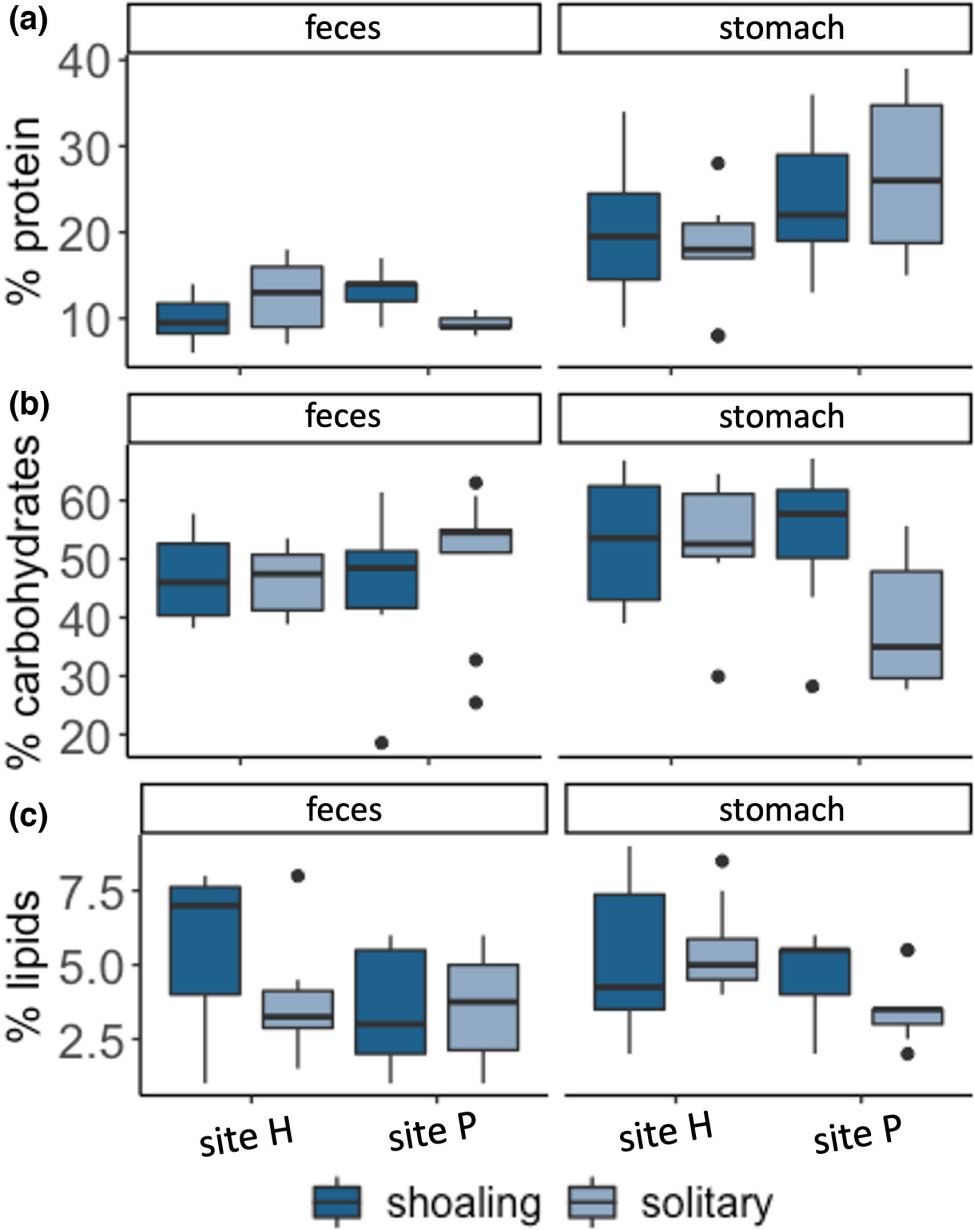
Percent dry matter of proteins (a), carbohydrates (b), and lipids (c) in the feces and stomach contents of shoaling and solitary *Acanthurus triostegus* from two backreef sites on Mo'orea

Protein (percentage of dry matter) for shoaling and solitary fish stomach contents is best predicted by the full model, which includes social behavior (shoaling or solitary), fish size (SL), site, and the interaction between site and social behavior as predictors (Figure 4a, Table 5). For the interaction of the site with social behavior, this model predicts a higher protein percentage for solitary fish at site P (+7.26), as well as higher protein for all fish at site P (+2.28) and larger fish (1.23), but lower protein percentage for just solitary fish, which is driven by the low protein percentage of solitary fish at site H (−2.79). The next best‐fit model (ΔAICc < 2) includes site, social behavior, and their interaction as predictors (Table A7). Protein percentage in shoaling and solitary *A. triostegus* feces is best predicted by a model that includes social behavior, site, and the interaction between site and social behavior as predictors (Figure 4a, Table 5). The next best‐fit model (ΔAICc < 2) includes site, social behavior, fish size, and the interaction between site and social behavior as predictors (Table A7).

**TABLE 5:** Best‐fit linear models for explaining variations in stomach content and feces macronutrients (proteins, carbohydrates, lipids) for shoaling and solitary *Acanthurus triostegus*

Coefficient	Protein				Carbohydrates				Lipids			
	Estimate	SE	*t*‐value	*p*‐value	Estimate	SE	*t*‐value	*p*‐value	Estimate	SE	*t*‐value	*p*‐value
Stomach												
Intercept	5.85	22.1	0.26	.79	105.14	29.8	3.52	.01	5.33	0.4	13.75	0
Social behavior (solitary)	−2.79	3.68	−0.76	.45	1.46	4.96	0.29	.77	‐	‐	‐	‐
SL (cm)	1.23	1.89	0.65	.52	‐4.45	2.54	−1.75	.09	‐	‐	‐	‐
Social: Site (solo:P)	7.26	5.37	1.35	.19	−19.17	7.24	−2.65	.01	‐	‐	‐	‐
Site (P)	2.28	3.69	0.62	.54	1.95	4.97	0.39	.06	1.22	0.6	−2.2	.03
Feces												
Intercept	10	0.85	11.81	0	88.54	35.5	2.49	.02	5.81	0.7	7.97	^0^
Social behavior (solitary)	2.44	1.23	1.99	.06	1.05	7.01	0.15	.88	−2.06	1	−2	.05
SL (cm)	‐	‐			3.51	2.96	−1.18	.25				
Social: Site (solo:P)	−6.37	1.74	−3.66	<.01	2.22	9.29	0.24	.81	2.23	1.4	1.59	.12
Site (P)	3.22	1.23	2.62	.01	−1.6	6.86	−0.23	.82	−2.48	1	−2.48	.02

*Note*: Protein pseudo‐*R* ^2^ = 0.18 (stomach), 0.29 (feces); carbohydrate pseudo‐*R* ^2^ = 0.35 (stomach), 0.10 (feces); lipids pseudo‐*R* ^2^ = 0.20 (stomach), 0.12 (feces).

Carbohydrate percentage in shoaling and solitary *A. triostegus* stomach is best predicted by a model that includes social behavior (shoaling or solitary), fish size (SL), site, and the interaction between site and social behavior as predictors (Figure 4b, Table 5). This model predicts a lower (−19.17) carbohydrate percentage for solitary fish at site P, as well as lower carbohydrate percentage for larger fish (−4.45), and a higher carbohydrate percentage for solitary fish (+1.46) and site P (+1.95). Carbohydrate percentage in shoaling and solitary *A. triostegus* feces is best predicted by a model that includes social behavior, site, the interaction between site and social behavior, and fish size as predictors (Figure 4b, Table 5).

Percentage of lipids in shoaling and solitary *A. triostegus* stomach is best predicted by a model that includes the site as a predictor (Figure 4c, Table 5). This model predicts a lower (−1.22) lipid percentage at site P. The next five best‐fit models (ΔAICc < 2) include: (1) site and fish size, (2) site, social behavior, and their interaction, (3) site and social behavior, (4) the full model (all predictors), and (5) site, social behavior, and fish size and predictors (Table A7). Lipid percentage in shoaling and solitary *A. triostegus* feces is best predicted by a model that includes social behavior, site, the interaction between site and social behavior, and fish size as predictors (Figure 4c, Table 5). The next best‐fit models (ΔAICc < 2) include: (1) social behavior, site, the interaction of site and social behavior, and fish size, (2) site and fish size, and (3) site, fish size, and social behavior as predictors (Table A7).

## 4 DISCUSSION

This study provides evidence that differences in the sociality of *A. triostegus* (i.e., shoaling versus solitary behavior modes) affect important attributes of their behavioral and functional ecology. We found that shoaling fish travel more linear distance and cover more total area than solitary conspecifics on the same reef. Additionally, shoaling fish and solitary fish graze in different areas as shoaling fish primarily graze within territories of herbivores, while solitary fish do not. Further, results from the stable isotope analyses suggest that these differences may be temporally durable, and results from the stable isotope and macronutrient analyses indicate that the dietary niche of shoaling fish may be more fixed than that of solitary fish.

We observed greater distance traveled and area covered (25‐min 95% KUD) by shoaling *A. triostegus* relative to their solitary counterparts (Figure 1a,b), as well as a higher proportion of territorial invasions by shoaling fish. Size of home and foraging ranges that vary with social behavior have been shown for other coral reef fish (Afonso et al., 2008) such as species of parrotfish where social behavior is separated into roving shoals, harems, or solitary territorial modes (Mumby & Wabnitz, 2002; Welsh & Bellwood, 2012a). While consistent with our results, some of these other fish species pose more challenging models to purely examine the role of sociality as their movement may be confounded by other complex behavioral interactions such as mating and reproductive behavior drivers. *A. triostegus* reproduction occurs on the reef crest in large spawning aggregations and therefore was possible to exclude as an interaction (Randall, 1961). Contrasting results have been observed for the relationship between gregarious behavior and movement. For example, in some parrotfish species, larger harems often have larger foraging ranges and territories than solitary fish that hold territories (Mumby & Wabnitz, 2002). However, *Acanthurus coeruleus*, a surgeonfish found on Caribbean reefs, exhibits similar variation in social behavior as *A. triostegus* in that it is found in solitary foraging modes and in large shoals; yet, solitary wandering *A. coeruleus* were found to traverse more distance and had larger foraging ranges than their shoaling conspecifics (Reinthal & Lewis, 1986). Further, on the Great Barrier Reef, shoaling *Scarus rivulatus*, an abundant parrotfish species, has similar home ranges when solitary and in shoals (Welsh & Bellwood, 2012a). Our observation of increased movement and larger areas covered by shoaling fish could be due to predation risk and resource availability. Predator avoidance can affect movement in fish, with fish at increased risk of predation opting to occupy smaller areas that provide structure for cover (Madin et al., 2010; Rooker et al., 2018). As shoaling can reduce predation risk, solitary *A. triostegus* may counter‐balance this risk by reducing movement. Further, results from a previous study on *A. triostegus* on Palmyra Atoll and Mo'orea found that solitary fish travel greater distance on Mo'orea, where natural predator abundance is lower, suggesting that movement may in part be influenced by predation (Guerra et al., 2022). Alternatively, if *A. triostegus* primarily use shoaling behavior to gain access to resources guarded by territorial herbivores, as is the case with the congener *A. coeruleus* (Reinthal & Lewis, 1986), movement may be dictated by the distribution of damselfish territories.

Territorial invasions by shoaling fish have been documented in other shoaling coral reef species (Catano et al., 2014; Dowdell et al., 2013; Foster, 1985), and for *A. triostegus* on other islands (Barlow, 1974). Algal farms maintained by the damselfish *S. nigricans*, in particular, received a high number of invasions by *A. triostegus* shoals. These damselfish territories are up to 3.4 times more productive than neighboring algal zones (Blanchette et al., 2019; Klumpp et al., 1987) and contain highly digestible red algae (Klumpp et al., 1987; Klumpp & Polunin, 1989) that is a desirable food source for surgeonfish including shoaling *A. triostegus* (Eurich et al., 2018).

The results from isotope and macronutrient analyses reflect our observed differences in the grazing by shoaling and solitary *A. triostegus*. While the standard deviation between sites and behavior types was considerable (Figure 3), the best‐fit model for δ^15^N isotope values suggests these behaviors may be more fixed beyond the short duration of our observational follows (Table 4). Differences in δ^15^N are usually attributed to differences in trophic level; however, the difference we observed (~0.3) is not large enough to indicate a shift in an entire trophic level (Post, 2002). Furthermore, *A. triostegus* is considered to be largely a herbivore (Abitia, 2011; Kelly et al., 2016; Nalley et al., 2021). Thus, this difference between solitary and shoaling fish is likely indicative of a different herbivorous dietary niche. A plausible explanation for the difference in δ^15^N isotope values is the tendency for shoals of *A. triostegus* to forage in *S. nigricans* territories. Damselfish territories promote higher epiphytal loads than those found outside of their territories (Ceccarelli, 2007; Ceccarelli et al., 2001; Jones et al., 2006) and δ^15^N isotope values signatures of macroalgae, and their epiphytes can differ (Hata & Umezawa, 2011; Yamamuro, 1999). Alternatively, δ^13^C isotope values were found to vary across sites and fish size but not between shoaling and solitary *A. triostegus*. Carbon isotopes are known to vary among species of marine plants and across space, supporting our findings of differing values for *A. triostegus* at the two sites (Carassou et al., 2008; Fry et al., 1982). Additionally, ontogeny has been shown to affect tissue δ^13^C values in *A. triostegus*, which may explain the relationship between fish size and δ^13^C (Frédérich et al., 2012). Interestingly, fish size was not a significant factor in our best‐fit δ^15^N model, despite the fact that δ^15^N is often even more strongly influenced by body size. Further, the Bayesian ellipses show a high overlap between shoaling fish, but not between solitary fish across the two sites, or between shoaling and solitary fish at either site (Figure 3b). This high overlap between shoaling fish supports our observation of a high proportion of foraging on damselfish territories by shoaling fish (Figure 1c), as *S. nigricans* territories are meticulously maintained and thus likely homogenous across sites (Blanchette et al., 2019; Hata & Kato, 2002). Solitary fish, however, are unable to access territories and their foraging may be more sensitive to resource availability across sites.

Results from the macronutrient analysis suggest that the observed differences in foraging between shoaling and solitary *A. triostegus* are nutritionally and ecologically consequential (Figure 4, Table 5). Stomach content reflects diet and can reveal nutritional intake (Mendes et al., 2018). Stomach content carbohydrate percentage varied with social behavior and site, suggesting initial nutritional intake is different across social groups. On the other hand, stomach content lipid percentage varied with the site (Figure 4, Table 5). There may be multiple explanations for this, including differences in resource availability across sites, and/or the nutritional composition of these resources. In line with the results from the Bayesian ellipses, the stomach content reveals that solitary fish seem to have a more variable diet within their social group and across sites (Figure 4, Table 5). The fecal nutrients indicate not only differences in diet but may also suggest differences in physiology between the two behavioral groups. Metabolic physiology and behavior are thought to be linked, where fish with higher metabolic rates tend to be more active and consume more food (Bailey et al., 2022; Killen et al., 2012; Metcalfe et al., 2016). Shoals and solitary fish may vary in their metabolic rates and nutrient assimilation, both of which could impact fecal nutrient composition. Importantly, the interaction between social behavior and site had the strongest effect on the concentration of protein in feces (Figure 4, Table 5).

The variation in the nutritional quality of feces across social groups may have ecological consequences. While it is known that herbivorous fish supply nutrients to their ecosystem through excretion and egestion (Allgeier et al., 2017), corals are sensitive to the ratios of nutrients supplied by fish (Allgeier et al., 2014), as well as the spatial scales of nutrient supply (Meyer et al., 1983). Both the difference in swimming behaviors and fecal nutrient concentrations suggest that shoaling and solitary fish may play different roles in nutrient recycling within coral reefs. Importantly, because we intentionally sampled macronutrients from individual fish that were most divergent in their stable isotope values, our macronutrient results may be best considered to provide insight into the upper bound differences between solitary and shoaling fish.

Heterospecific fish are associated with almost all *A. triostegus* shoals on both Palmyra Atoll and Mo'orea (Table A3). Associations of predatory and nonpredatory fish with shoals of *A. triostegus* have been previously documented (Barlow, 1974; Madin & Madin, 2011). We observed more predators associated with shoals on Palmyra Atoll than on Mo'orea, as well as more higher‐trophic level fish associated with shoals on Palmyra Atoll. Mo'orea hosts a smaller predator biomass than Palmyra Atoll due to a history of commercial and subsistence fishing, which likely explains the observed difference between the two islands (Davis et al., 2017). Piscivores often associate with fish shoals to approach prey by using the focal shoaling species as cover (Lukoschek & McCormick, 2002), and may opportunistically prey on the focal shoaling species (Pers. Obs). Species such as *Lutjanus bohar*, *Caranx melampygus*, and *Aulostomus chinensis*, for example, will associate with shoals of surgeonfish and approach territorial damselfish that may be temporarily preoccupied with defending their territory from shoaling herbivores (Madin & Madin, 2011; Ormond, 2009). Similarly, heterospecific invertivore and herbivorous fishes may associate with shoals to accrue benefits such as gaining access to foraging on algae or invertebrates within damselfish territories (Alevizon, 1976; Klumpp & Polunin, 1989; Lukoschek & McCormick, 2002; Montgomery, 1981; Ormond, 2009). The high proportion of invasions of herbivorous fish territories by shoaling fish supports the hypothesis that shoals may traverse long distances in search of heterospecific fish territories and that nonterritorial heterospecific fish may associate with shoals to gain access to these areas. Additionally, mixed‐species grouping is sometimes thought to provide a trade‐off in benefits to the focal species involved, such as increased protection through vigilant behavior by associated species (Paijmans et al., 2019). The benefits to the associated herbivores and piscivores are clear; however, without further study, it is not possible to conclusively determine whether *A. triostegus* accrue any benefits from these associations.

Collectively, our results provide an important starting point to better understand the ecological role of the two social modes. There are important limitations to our study that must be considered. For example, our study explored differences only between two islands, and fish were collected from two sites on a single island, thus it is possible that environmental factors beyond *A. triostegus* social behavior may influence our observations and results. For example, differences in habitat structure and resource availability can influence fish movement (Tootell & Steele, 2016) and diet (Francini‐Filho et al., 2010). Additionally, by design, our observational follows took place on the extremes of social behavior—large shoals and individual fish. Future studies should include a range of shoal sizes, to better assess the point at which the differences we observed and measured begin to emerge or whether these differences vary by group size. Finally, our behavioral observations were limited in duration and do not account for the activity or these fish throughout a full day, where behavioral social modes may shift. However, results from the stable isotope analysis and our preliminary fish resighting data (Appendix B) suggest these behavioral social modes may be fixed and long‐lasting (e.g., at least 3 years). Further and more extensive investigation is necessary to confirm these observations and to test their applicability in different contexts.

The social behavior of fish may be subject to alteration in a fished ocean, leading to a reduction in shoaling and schooling behavior (Guerra et al., 2020, 2022; Sbragaglia et al., 2021). We previously showed, for example, that shoaling behavior in *A. triostegus*, in particular, may be shifting towards fewer schools on Mo'orea, where natural predator populations have been depleted through fishing (Guerra et al., 2022). This work extends the significance of those findings by suggesting that the functional role and resultant ecological influence of *A. triostegus* on a coral reef are likely to change as a result of such shifts in social behavior.

With this new information on how shoaling behavior may shape coral reef ecosystems, we identify three of many potential ecosystem‐wide impacts of a shift in *A. triostegus* social behavior towards fewer shoaling fish (and more solitary fish):

### 4.1 Reduction in grazing within territories

Considering the high (80%–90%) proportion of shoaling *A. triostegus* grazing that occurred within the territory of heterospecific fish species, a shift towards more solitary fish would likely reduce the amount of grazing occurring in these areas (Figure 1c). As solitary fish are mostly unable to access these well‐defended areas, a decrease in shoaling behavior would correspond to a decrease in the total amount of grazing within these territories. Reductions in grazing within *S. nigricans* territories due to reductions in shoaling behavior may have important outcomes for coral reefs and could influence overall coral‐algal dynamics on these reefs. Whether territorial damselfish algal gardens are beneficial or detrimental to coral reef health remains unresolved and their effect is likely context‐dependent, as studies have found that territories can (1) serve as refuges for macroalgae, which could facilitate phase shifts towards algae‐dominated systems (Hoey & Bellwood, 2010), (2) cause decreases in coral survival and reduced coral health (Arnold et al., 2010; Casey et al., 2014; Potts, 1977; Vermeij et al., 2015), and (3) cause increases in coral survival within damselfish territories (Gochfeld, 2010). Importantly, in areas where damselfish territories are detrimental to reef health by allowing macroalgae to outcompete live coral, a substantial reduction in grazing within territories may promote phase shifts to algae‐dominated systems.

### 4.2 Reduced subsidies to heterospecific fish associates

Heterospecific piscivores and herbivores were found associated with *A. triostegus* shoals on both islands. Although our study did not compare predation success or foraging of these heterospecific fish while associated versus unassociated with shoals, studies suggest that these associations commonly confer benefits to the associated fish (Aronson, 1983; Ormond, 2009). If foraging alongside *A. triostegus* shoals facilitates a high proportion of the dietary needs of associated heterospecifics, a decrease in shoals might lead to dietary shifts, dietary quality, and possibly survival or health of individuals. Notably, these heterospecific fish include species of herbivores that also play important roles in mediating coral‐macroalgae interactions (Mumby et al., 2006). Future studies will be required to properly explore the functional outcomes of these interspecific dynamics.

### 4.3 Shifts in spatial distribution and composition of bioavailable nutrient supply

Consumers on coral reefs can influence their environment not just through grazing but through supplying nutrients via egestion and excretion, providing nutrients to both macroalgae and corals (Allgeier et al., 2017; Burkepile et al., 2013; Munsterman et al., 2021). Nutrient supply from fish can be detrimental to reefs by facilitating macroalgae growth, or beneficial by fostering coral growth (Burkepile et al., 2013; Meyer et al., 1983). In instances where fish nutrient supply facilitates coral growth, coral can be sensitive to ratios of nutrients supplied by fish (Allgeier et al., 2014). Shoaling fish maintain a fixed dietary niche across sites, likely because of foraging within *S. nigricans* territories, but solitary fish appear to shift their diet based on local resource availability at each site (Figure 4). Thus, a shift towards a solitary social mode may increase variability in nutrient supply. Additionally, the spatial scale at which nutrients are supplied can also influence productivity and coral growth. For example, corals that shelter fish schools experience more growth due to the concentrated pulses in nutrients, as opposed to those that only experienced sporadic nutrient supply (Shantz et al., 2015). We did not measure defecation by *A. triostegus* shoals, and whether defecation was “pulsed” by all members of a shoal simultaneously, or whether fish defecated at different times. However, as shoals traverse larger extents of the reef than solitary fish, their effect on nutrient supply becomes distributed over larger areas.

Overall, our study suggests that the social behavior of individuals plays an important role in mediating their ecological function. Aggregating wildlife that plays pivotal ecological roles is found across various ecosystems, including annual wildebeest migrations that contribute significantly to river nutrient cycling due to mass drownings (Subalusky et al., 2017), colonial nesting seabirds that provide marine‐derived nutrients to oceanic islands (Ellis et al., 2006), and herding ungulates that can alter nutrient cycling and plant community composition through grazing, trampling, and defecation (Hobbs, 1996). Given the ubiquity of these social behaviors and that such behaviors may be subject to alteration from human disturbance, more attention and future work should be dedicated to better understanding the relationship between animal sociality and ecological function.

## AUTHOR CONTRIBUTIONS

**Ana Sofia Guerra:** Conceptualization (lead); data curation (lead); formal analysis (lead); funding acquisition (equal); investigation (lead); methodology (lead); project administration (lead); resources (lead); visualization (lead); writing – original draft (lead). **Jacey C. Van Wert:** Data curation (equal); formal analysis (equal); methodology (equal); writing – review and editing (equal). **Alison J. Haupt:** Investigation (equal); methodology (equal); writing – review and editing (equal). **Douglas J. McCauley:** Conceptualization (supporting); formal analysis (supporting); methodology (supporting); supervision (supporting); writing – review and editing (supporting). **Erika J. Eliason:** Funding acquisition (equal); methodology (supporting); resources (supporting); writing – review and editing (equal). **Hillary S. Young:** Formal analysis (supporting); investigation (supporting); methodology (supporting); writing – review and editing (equal). **David Lecchini:** Funding acquisition (equal); project administration (equal); resources (equal); writing – review and editing (equal). **Tim D. White:** Conceptualization (equal); investigation (equal); methodology (equal); writing – review and editing (equal). **Jennifer E. Caselle:** Conceptualization (supporting); formal analysis (supporting); funding acquisition (equal); methodology (supporting); resources (equal); supervision (equal); writing – review and editing (equal).

## FUNDING INFORMATION

JEC was funded by the Marisla Foundation. This is PARC publication #0163.

## Supporting information


FigureA1

FigureA2

FigureA3

FigureA4

FigureA5

Appendix S1

## Data Availability

Data and site descriptions are available at: https://doi.org/10.6073/pasta/678b0008e9906e402bcccde906fbbf25.

## APPENDIX A

**TABLE A1:** Species list for heterospecific fish associated with *Acanthurus triostegus* on Palmyra Atoll and Mo'orea

	Palmyra Atoll	Mo'orea
Predatory	*Aulostomus chinensis*	*Aulostomus chinensis*
	*Caranx melampygus*	*Fistularia commersoni*
	*Carcharhinus melanopterus*	*Caranx melampygus*
	*Cephalopholis argus*	
	*Lutjanus bohar*	
Non‐predatory	*Acanthurus blochii*	*Acanthurus guttatus*
	*Acanthurus xanthoptherus*	*Cantherhines dumerilii*
	*Chlorurus frontalis*	*Chlorurus spilurus*
	*Chlorurus spilurus*	*Scarus psittacus*
	*Kyphosis sp*.	*Siganus argenteus*
	*Mellycthis niger*	
	*Scarus altipinnis*	
	*Scarus frenatus*	
	*Scarus oviceps*	
	*Scarus rubroviolaceus*	

**TABLE A2:** Predictors and interactions tested for finding best‐fit models in analyses

Analysis	Fixed effects	Random effects
Linear mixed model (distance traveled, 95% KUD, territorial invasions, non‐predatory and predatory fish associations)	Social behavior	Island
		Site (within island)
		Time of day
Linear models (stable isotopes, macronutrients)	Social behavior	‐
	Size (SL)	
	Site	
	Social behavior * site	

**TABLE A3:** Best fit linear mixed models for explaining variation in distance traveled and 95% KUD for shoaling *Acanthurus triostegus*

	Distance traveled			95% KUD		
Fixed effect	Estimate	SE	*t*‐value	Estimate	SE	*t*‐value
Intercept	9.35	2.05	4.55	3.95	0.23	17.36
Shoal size	‐	‐	‐	‐	‐	‐
**Random effect**	**Variance**	**SD**		**Variance**	**SD**	
Time of day	1.78	1.33		0.00	0.06	
Island	6.42	2.53		0.04	0.21	
Site (within island)	0.33	0.57		0.23	0.48	

**TABLE A4:** Summary of mean and standard deviation (SD) of proportion of time spent grazing and grazing within heterospecific territories for shoals and solitary *Acanthurus triostegus*, and proportion of all follows during which predatory and non‐predatory fish were associated.

Observation		Shoals		Solitary	
		Mean	SD	Mean	SD
Grazing (proportion of follow)	Palmyra Atoll	0.58	0.17	0.51	0.24
	Mo'orea	0.62	0.14	0.6	0.17
Territorial invasions (proportion of grazing events)	Palmyra Atoll	0.9	0.12	0.02	0.04
	Mo'orea	0.83	0.16	0.13	0.14
Non‐predatory fish associations (proportion of all follows)	Palmyra Atoll	0.95	0.23	0	0
	Mo'orea	0.71	0.46	0	0
Predatory fish associations (proportion of all follows)	Palmyra Atoll	1	0	0	0
	Mo'orea	0.24	0.44	0	0

**TABLE A5:** Wilcoxon‐rank sum test results for comparing *Acanthurus triostegus* size between the two collection sites and two social modes (shoaling and solitary)

	W	*p*‐value
Sites	1280	.21
Social behavior	1413.5	.95

**TABLE A6:** Best fit linear models for explaining variation in δ15N and δ13C stable isotope values of muscle tissue of shoaling and solitary *Acanthurus triostegus*

δ15N					δ13C^a^			
Coefficient	Estimate	SE	*t*‐value	*p*‐value	Estimate	SE	*t*‐value	*p*‐value
Intercept	4.87	0.67	7.26	.00	−5.97	1.08	−5.58	.00
Social behavior (solitary)	0.35	0.1	3.43	.001				
SL (cm)	0.15	0.06	2.64	.01	−0.45	0.10	−5.58	.00
Site (P)					−0.54	0.16	−2.24	.03

^a^ These models include all size fish.

**TABLE A7:** Next best‐fit (ΔAICc < 2) linear models for explaining variations in stomach content and fecal macronutrients (proteins and lipids) for shoaling and solitary *A. triostegus*. Best fit model presented in main text

	Coefficient	protein				lipids			
		Estimate	SE	*t*‐value	*p*‐value	Estimate	SE	*t*‐value	*p*‐value
Stomach 1	Intercept	20.10	2.48	8.11	.00	−1.66	4.26	−0.39	.70
	Social behavior (solitary)	−2.43	3.61	−0.68	.5	‐	‐	‐	‐
	SL (cm)	‐	‐	‐	‐	0.59	0.36	1.65	.11
	Social:site (solo:P)	6.26	5.1	1.23	.23	‐	‐	‐	‐
	Site (P)	2.68	3.6	0.74	.46	−1.11	0.55	−2.04	.05
Stomach 2	Intercept					5.15	0.54	9.46	.00
	Social behavior (solitary)					0.35	0.77	0.45	.65
	SL (cm)					‐	‐	‐	‐
	Social:site (solo:P)					−1.52	1.10	−1.38	.18
	Site (P)					−0.43	0.79	−0.54	.59
Stomach 3	Intercept					5.52	0.48	11.51	.00
	Social behavior (solitary)					−0.39	0.56	−0.70	.49
	SL (cm)					−	−	−	−
	Social:site (solo:P)					−	−	−	−
	Site (P)					−1.21	0.56	−2.70	.04
Stomach 4	Intercept					−0.35	1.47	−0.08	.94
	Social behavior (solitary)					0.13	0.78	0.17	.87
	SL (cm)					0.47	0.38	1.24	.22
	Social:site (solo:P)					−1.06	1.16	−0.91	.37
	Site (P)					−0.58	0.80	−0.73	.47
Stomach 5	Intercept					1.43	4.30	−0.33	.74
	Social behavior (solitary)					−0.38	0.55	−69.00	.49
	SL (cm)					0.59	0.36	1.63	.11
	Social:site (solo:P)					−	−	−	−
	Site (P)					−1.10	0.55	−2.01	.05
Feces 1	Intercept	8.23	7.35	11.12	.27	13.34	5.68	2.35	.03
	Social behavior (solitary)	2.36	1.3	1.81	.08	−1.71	1.05	−1.62	.12
	SL (cm)	0.15	0.63	0.24	.81	−0.65	0.49	−1.34	.19
	Social:site (solo:P)	−6.19	1.9	−3.27	.01	1.53	1.48	1.03	.31
	Site (P)	3.17	1.26	2.51	.02	−2.25	1.01	−2.24	.03
Feces 2	Intercept					14.45	5.48	2.64	.01
	Social behavior (solitary)					‐	‐	‐	‐
	SL (cm)					−0.81	0.46	−1.77	.09
	Social:site (solo:P)					‐	‐	‐	‐
	Site (P)					−1.52	0.70	−2.16	.04
Feces 3	Intercept					15.04	5.45	2.76	.01
	Social behavior (solitary)					−0.88	0.69	−1.28	.21
	SL (cm)					−0.83	0.46	−1.82	.08
	Social:site (solo:P)					‐	‐	‐	‐
	Site (P)					−1.50	0.70	−2.15	.04

## APPENDIX B

### B.1 Surveys of benthic cover at various scales

To understand how variation in foraging behavior may have been influenced by variation in benthic habitats across and within islets we utilized existing benthic data (Palmyra Atoll collected in 2006) supplemented with new benthic data collected with identical methods by the same observers at Mo'orea Island (2022). In brief cover was surveyed across a series of 16 square quadrats (each 1 m^2^) spaced every 5 m along a 100 m belt transect, with the transect typically oriented parallel to the reef crest. For the inter‐island comparisons, a total of 13 backreef sites were used (Palmyra Atoll = 7, Mo'orea = 6) with the values of all 16 quadrats pooled at the site level prior to analysis. Given the very small spatial scale of study sites within Mo'orea, for the intra island comparison at Mo'orea the quadrats are instead treated as independent replicates (n = 16 per site). This benthic data was collected asynchronously with fish survey data and not at the same time at two sites limiting strength of comparisons. However, while these differences in temporal sampling might be expected to exacerbate any underlying differences in benthic habitat analysis showed no statistically significant differences within any type of cover across islands although there were trends for more live coral and CCA at Palmyra Atoll as compared to Mo'orea. This tendency could, however, be an artifact of temporal differences in sampling, noting that there were significant coral die off events in both regions, but particularly French Polynesia, in 2019.

## APPENDIX C

### C.1 Resighting of *Acanthurus triostegus*

*Acanthurus triostegus* resight surveys were conducted at a single site on Mo'orea, French Polynesia (17°28'47.1"S 149°47'37.1"W). A total of 12 surveys were conducted between 29‐September‐2019 and 22‐October‐2019. Any shoals and solitary fish were photographed for later analysis. Towards later surveys, individual solitary and paired fish were easily identifiable by observer and their presence was logged without photographing.

Shoaling and solitary fish in photographs were identified using right‐side markings only (Figure A4). We identified and re‐sighted five solitary fish and seven shoaling fish. Every resighted fish was exhibiting social behavior (shoaling or solitary) across sightings. Average number of resights was 3.5 ± 2.2, with a maximum of 8 resights and a minimum of 2. The mean time span between first and last resight was 14.7 ± 6.5 days, with a maximum of 20 days and a minimum of 1 day. We also photographed putative matches for 2 solitary and 2 shoaling fish in the same location and same behavioral mode 21.5 months later; and 2 of the same solitary fish in the same behavioral mode 36 months later. However, the shoaling fish observed at the 21.5 months observation point exhibited some subtle growths to their melanistic patterns, as such without knowledge on how these patterns may change over time, we cannot provide complete certainty that these are the same fish (Figure A4). Future work can help further substantiate if this behavioral fidelity does indeed persist for long time periods and whether this pattern remains consistent across a wider range of geographic sites.
