# Supplementary figures and images for: Differences in the behavior and diet between shoaling and solitary surgeonfish (Acanthurus triostegus)
Source: Ecol Evol. 2023 Jan 6;13(1):e9686. doi: 10.1002/ece3.9686 (PMC9817200; doi:10.1002/ece3.9686)

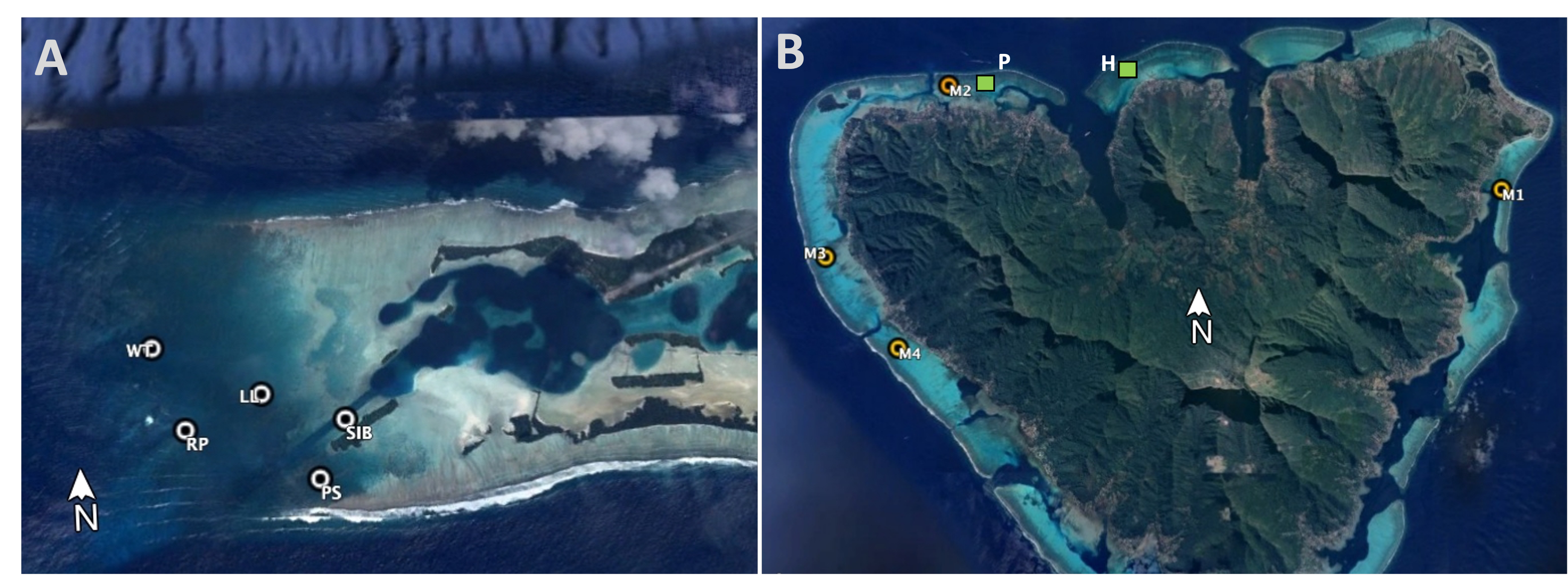

Supplement: Supplementary file 1 — FigureA1 [file ECE3-13-e9686-s001.tiff]

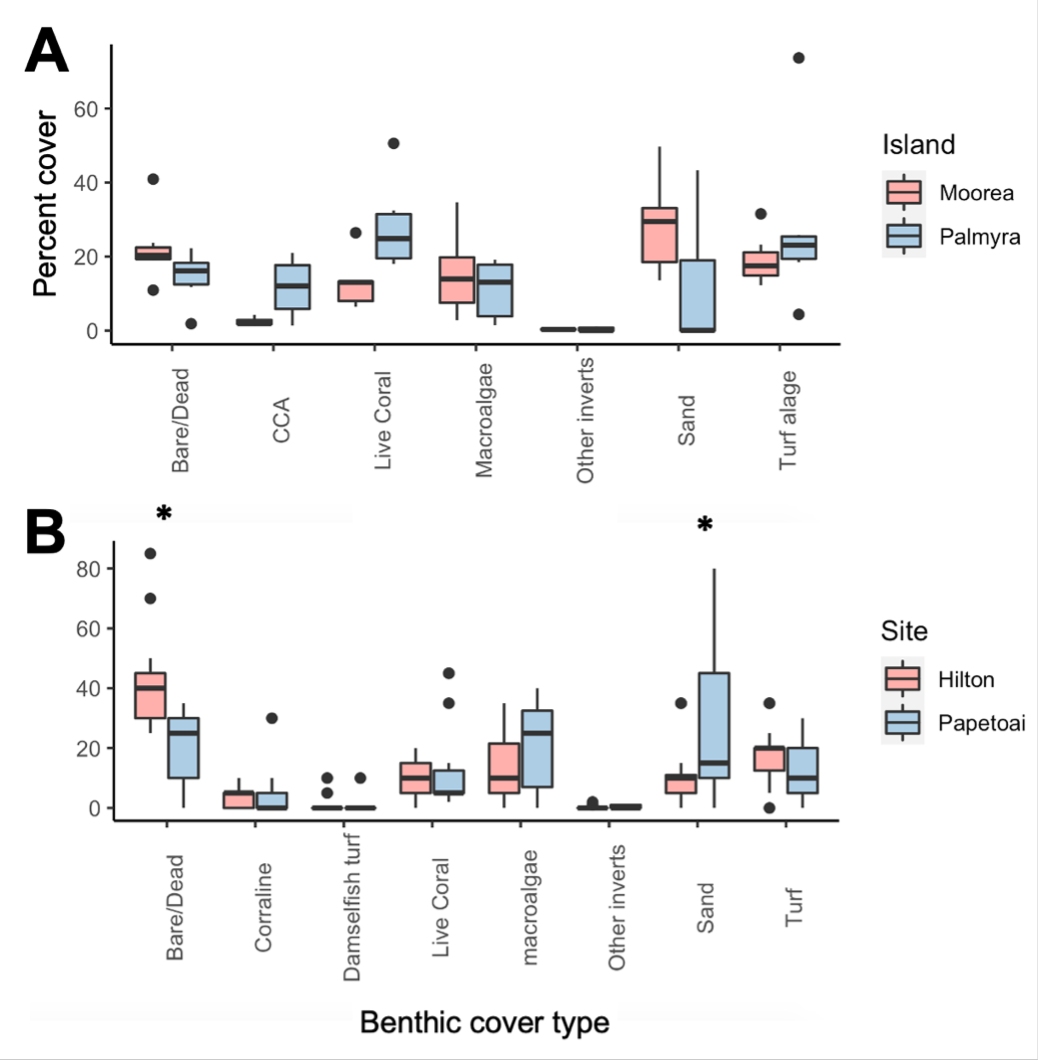

Supplement: Supplementary file 2 — FigureA2 [file ECE3-13-e9686-s005.tiff]

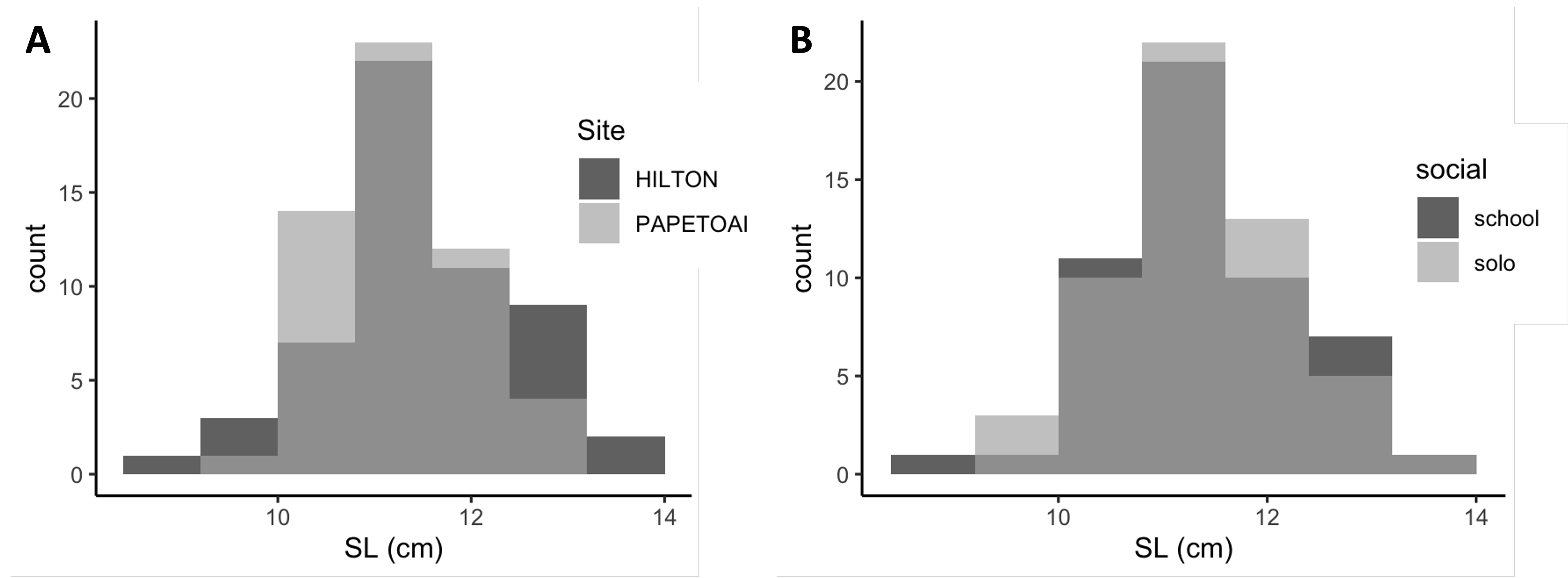

Supplement: Supplementary file 3 — FigureA3 [file ECE3-13-e9686-s002.tiff]

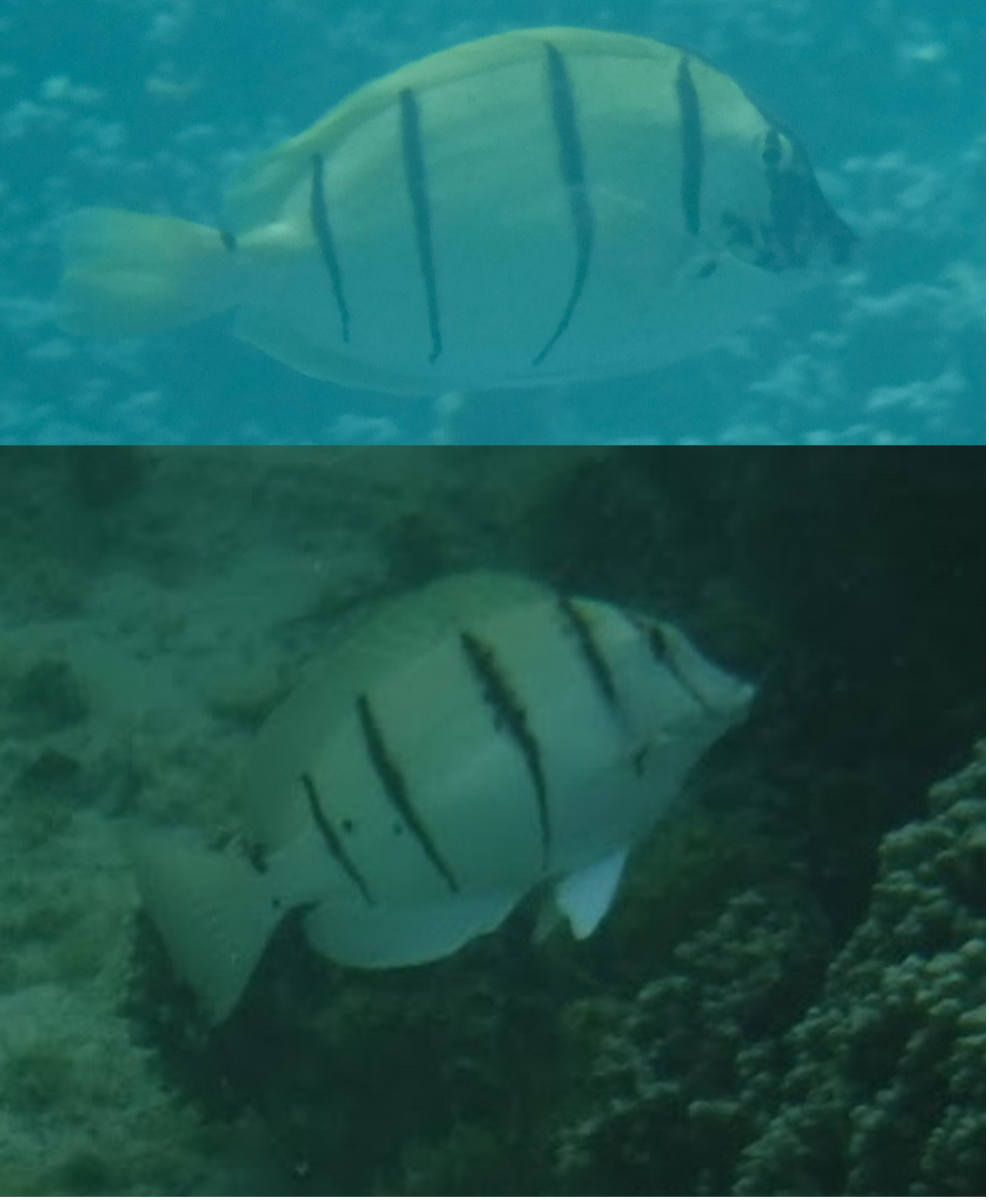

Supplement: Supplementary file 4 — FigureA4 [file ECE3-13-e9686-s006.tiff]

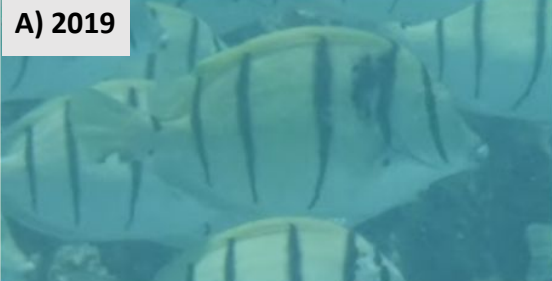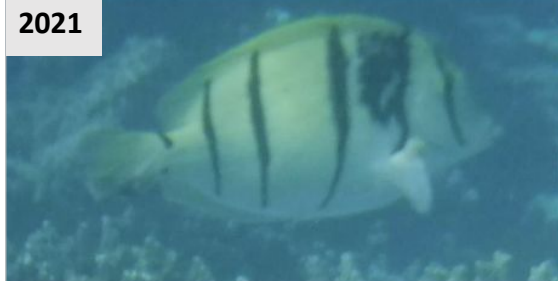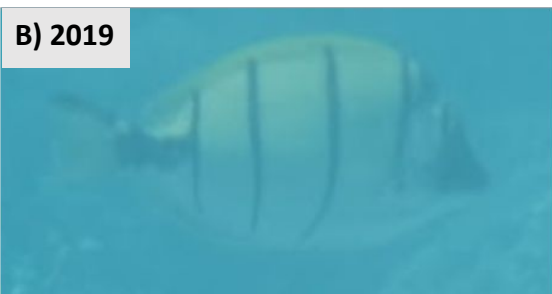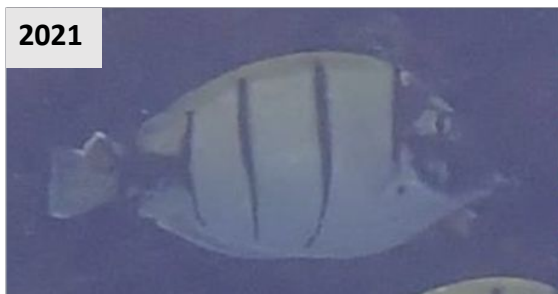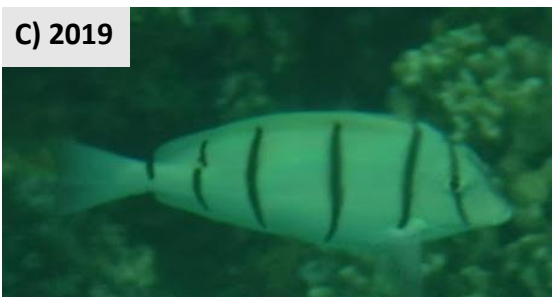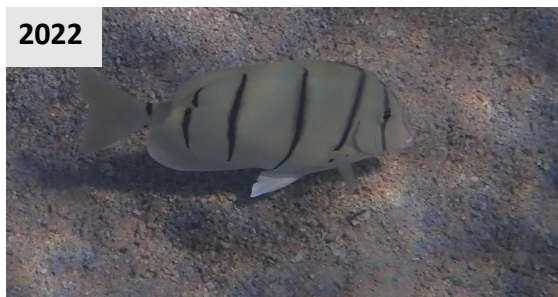

Supplement: Supplementary file 5 — FigureA5 [file ECE3-13-e9686-s003.pdf]
