# Supplementary material for: Differences in the behavior and diet between shoaling and solitary surgeonfish (Acanthurus triostegus)
Source: Ecol Evol. 2023 Jan 6;13(1):e9686. doi: 10.1002/ece3.9686 (PMC9817200; doi:10.1002/ece3.9686)
Supplement: Supplementary file 6 — Appendix S1 [file ECE3-13-e9686-s004.docx]

Appendix

**Figure A1.** Field sites on (A) Palmyra Atoll, USA and (B) Mo’orea, French Polynesia. Circles represent behavioral observation sites, and squares (sites P and H, only on Mo’orea (B), represent sampling sites). GPS Coordinates for sites can be found in data repository at https://doi.org/10.25349/D94617

**Figure A2.** Benthic cover surveys (described in Text A2) categories revealed (A) no significant differences within any cover type either between Mo’orea Island and Palmyra Atoll but (B) a lower amount of bare rock/dead coral and higher amount of sand in Papetoai between focal sites within Mo’orea Island (significant differences marked with an asterisk)

**Figure A3.** Histograms of sampled fish sizes (standard length in centimeters) across the two sites on Mo’orea, French Polynesia (A), and distribution across the two social modes: shoaling and solitary (B).

| **Table A1.** Species list for heterospecific fish associated with *A. triostegus* on Palmyra Atoll and Mo’orea | | |
| --- | --- | --- |
|  | **Palmyra Atoll** | **Mo’orea** |
| Predatory | *Aulostomus chinensis* | *Aulostomus chinensis* |
|  | *Caranx melampygus* | *Fistularia commersoni* |
|  | *Carcharhinus melanopterus* | *Caranx melampygus* |
|  | *Cephalopholis argus* |  |
|  | *Lutjanus bohar* |  |
| Non-predatory | *Acanthurus blochii* | *Acanthurus guttatus* |
|  | *Acanthurus xanthoptherus* | *Cantherhines dumerilii* |
|  | *Chlorurus frontalis* | *Chlorurus spilurus* |
|  | *Chlorurus spilurus* | *Scarus psittacus* |
|  | *Kyphosis sp.* | *Siganus argenteus* |
|  | *Mellycthis niger* |  |
|  | *Scarus altipinnis* |  |
|  | *Scarus frenatus* |  |
|  | *Scarus oviceps* |  |
|  | *Scarus rubroviolaceus* |  |

| **Table A2.** Predictors and interactions tested for finding best-fit models in analyses | | |
| --- | --- | --- |
| **Analysis** | **Fixed effects** | **Random effects** |
| linear mixed model (distance traveled, 95% KUD, territorial invasions, non-predatory and predatory fish associations) | social behavior | island |
|  |  | site (within island) |
|  |  | time of day |
| linear models (stable isotopes, macronutrients) | social behavior | - |
|  | size (SL) |  |
|  | site |  |
|  | social behavior * site |  |

| **Table A3.** Best fit linear mixed models for explaining variation in distance traveled and 95% KUD for shoaling *Acanthurus triostegus*. | | | | | | |
| --- | --- | --- | --- | --- | --- | --- |
|  | **distance traveled** | | | **95% KUD** | | |
| Fixed effect | estimate | SE | t-value | estimate | SE | t-value |
| intercept | 9.35 | 2.05 | 4.55 | 3.95 | 0.23 | 17.36 |
| shoal size | - | - | - | - | - | - |
| Random effect | variance | SD |  | variance | SD |  |
| time of day | 1.78 | 1.33 |  | 0.00 | 0.06 |  |
| island | 6.42 | 2.53 |  | 0.04 | 0.21 |  |
| site (within island) | 0.33 | 0.57 |  | 0.23 | 0.48 |  |

| **Table A4.** Summary of mean and standard deviation (SD) of proportion of time spent grazing and grazing within heterospecific territories for shoals and solitary Acanthurus triostegus, and proportion of all follows during which predatory and non-predatory fish were associated. | | | | | |
| --- | --- | --- | --- | --- | --- |
| observation |  | shoals | | solitary | |
|  |  | mean | SD | mean | SD |
| grazing (proportion of follow) | Palmyra Atoll | 0.58 | 0.17 | 0.51 | 0.24 |
|  | Mo’orea | 0.62 | 0.14 | 0.6 | 0.17 |
| territorial invasions (proportion of grazing events) | Palmyra Atoll | 0.9 | 0.12 | 0.02 | 0.04 |
|  | Mo’orea | 0.83 | 0.16 | 0.13 | 0.14 |
| non-predatory fish associations (proportion of all follows) | Palmyra Atoll | 0.95 | 0.23 | 0 | 0 |
|  | Mo’orea | 0.71 | 0.46 | 0 | 0 |
| predatory fish associations (proportion of all follows) | Palmyra Atoll | 1 | 0 | 0 | 0 |
|  | Mo’orea | 0.24 | 0.44 | 0 | 0 |

| **Table A5**. Wilcoxon-rank sum test results for comparing A. triostegus size between the two collection sites and two social modes (shoaling and solitary) | | |
| --- | --- | --- |
|  | W | p-value |
| Sites | 1280 | 0.21 |
| Social behavior | 1413.5 | 0.95 |

| **Table A6.** Best fit linear models for explaining variation in δ15N and δ13C stable isotope values of muscle tissue of shoaling and solitary Acanthurus triostegus* | | | | | | | | |
| --- | --- | --- | --- | --- | --- | --- | --- | --- |
| δ15N | | | | | δ13C | | | |
| coefficient | estimate | SE | t-value | p-value | estimate | SE | t-value | p-value |
| intercept | 4.87 | 0.67 | 7.26 | 0.00 | -5.97 | 1.08 | -5.58 | 0.00 |
| social behavior (solitary) | 0.35 | 0.1 | 3.43 | 0.001 |  |  |  |  |
| SL (cm) | 0.15 | 0.06 | 2.64 | 0.01 | -0.45 | 0.10 | -5.58 | 0.00 |
| site (P) |  |  |  |  | -0.54 | 0.16 | -2.24 | 0.03 |
| *these models include all size fish | | | | | | | | |

| **Table A7.** Next best-fit (ΔAICc <2) linear models for explaining variations in stomach content and fecal macronutrients (proteins and lipids) for shoaling and solitary *A. triostegus*. Best fit model presented in main text. | | | | | | | | | | | |
| --- | --- | --- | --- | --- | --- | --- | --- | --- | --- | --- | --- |
|  |  | **protein** | | | | **lipids** | | | | |  |
|  | coefficient | estimate | SE | t-value | p-value | estimate | SE | t-value | p-value |  |  |
| stomach 1 | intercept | 20.10 | 2.48 | 8.11 | 0.00 | -1.66 | 4.26 | -0.39 | 0.70 |  |  |
|  | social behavior (solitary) | -2.43 | 3.61 | -0.68 | 0.5 | - | - | - | - |  |  |
|  | SL (cm) | - | - | - | - | 0.59 | 0.36 | 1.65 | 0.11 |  |  |
|  | social:site (solo:P) | 6.26 | 5.1 | 1.23 | 0.23 | - | - | - | - |  |  |
|  | site (P) | 2.68 | 3.6 | 0.74 | 0.46 | -1.11 | 0.55 | -2.04 | 0.05 |  |  |
| stomach 2 | intercept |  |  |  |  | 5.15 | 0.54 | 9.46 | 0.00 |  |  |
|  | social behavior (solitary) |  |  |  |  | 0.35 | 0.77 | 0.45 | 0.65 |  |  |
|  | SL (cm) |  |  |  |  | - | - | - | - |  |  |
|  | social:site (solo:P) |  |  |  |  | -1.52 | 1.10 | -1.38 | 0.18 |  |  |
|  | site (P) |  |  |  |  | -0.43 | 0.79 | -0.54 | 0.59 |  |  |
| stomach 3 | intercept |  |  |  |  | 5.52 | 0.48 | 11.51 | 0.00 |  |  |
|  | social behavior (solitary) |  |  |  |  | -0.39 | 0.56 | -0.70 | 0.49 |  |  |
|  | SL (cm) |  |  |  |  | - | - | - | - |  |  |
|  | social:site (solo:P) |  |  |  |  | - | - | - | - |  |  |
|  | site (P) |  |  |  |  | -1.21 | 0.56 | -2.70 | 0.04 |  |  |
| stomach 4 | intercept |  |  |  |  | -0.35 | 1.47 | -0.08 | 0.94 |  |  |
|  | social behavior (solitary) |  |  |  |  | 0.13 | 0.78 | 0.17 | 0.87 |  |  |
|  | SL (cm) |  |  |  |  | 0.47 | 0.38 | 1.24 | 0.22 |  |  |
|  | social:site (solo:P) |  |  |  |  | -1.06 | 1.16 | -0.91 | 0.37 |  |  |
|  | site (P) |  |  |  |  | -0.58 | 0.80 | -0.73 | 0.47 |  |  |
| stomach 5 | intercept |  |  |  |  | 1.43 | 4.30 | -0.33 | 0.74 |  |  |
|  | social behavior (solitary) |  |  |  |  | -0.38 | 0.55 | -69.00 | 0.49 |  |  |
|  | SL (cm) |  |  |  |  | 0.59 | 0.36 | 1.63 | 0.11 |  |  |
|  | social:site (solo:P) |  |  |  |  | - | - | - | - |  |  |
|  | site (P) |  |  |  |  | -1.10 | 0.55 | -2.01 | 0.05 |  |  |
| feces 1 | intercept | 8.23 | 7.35 | 11.12 | 0.27 | 13.34 | 5.68 | 2.35 | 0.03 |  |  |
|  | social behavior (solitary) | 2.36 | 1.3 | 1.81 | 0.08 | -1.71 | 1.05 | -1.62 | 0.12 |  |  |
|  | SL (cm) | 0.15 | 0.63 | 0.24 | 0.81 | -0.65 | 0.49 | -1.34 | 0.19 |  |  |
|  | social:site (solo:P) | -6.19 | 1.9 | -3.27 | 0.01 | 1.53 | 1.48 | 1.03 | 0.31 |  |  |
|  | site (P) | 3.17 | 1.26 | 2.51 | 0.02 | -2.25 | 1.01 | -2.24 | 0.03 |  |  |
| feces 2 | intercept |  |  |  |  | 14.45 | 5.48 | 2.64 | 0.01 |  |  |
|  | social behavior (solitary) |  |  |  |  | - | - | - | - |  |  |
|  | SL (cm) |  |  |  |  | -0.81 | 0.46 | -1.77 | 0.09 |  |  |
|  | social:site (solo:P) |  |  |  |  | - | - | - | - |  |  |
|  | site (P) |  |  |  |  | -1.52 | 0.70 | -2.16 | 0.04 |  |  |
| feces 3 | intercept |  |  |  |  | 15.04 | 5.45 | 2.76 | 0.01 |  |  |
|  | social behavior (solitary) |  |  |  |  | -0.88 | 0.69 | -1.28 | 0.21 |  |  |
|  | SL (cm) |  |  |  |  | -0.83 | 0.46 | -1.82 | 0.08 |  |  |
|  | social:site (solo:P) |  |  |  |  | - | - | - | - |  |  |
|  | site (P) |  |  |  |  | -1.50 | 0.70 | -2.15 | 0.04 |  |  |

**Text A1: Surveys of benthic cover at various scales**

To understand how variation in foraging behavior may have been influenced by variation in benthic habitats across and within islets we utilized existing benthic data (Palmyra Atoll collected in 2006) supplemented with new benthic data collected with identical methods by the same observers at Mo’orea Island (2022). In brief cover was surveyed across a series of 16 square quadrats (each 1m^2^) spaced every 5 m along a 100 m belt transect, with the transect typically oriented parallel to the reef crest. For the inter-island comparisons, a total of 13 backreef sites were used (Palmyra Atoll = 7, Mo’orea =6) with the values of all 16 quadrats pooled at the site level prior to analysis. Given the very small spatial scale of study sites within Mo’orea, for the intra island comparison at Mo’orea the quadrats are instead treated as independent replicates (n = 16 per site). This benthic data was collected asynchronously with fish survey data and not at the same time at two sites limiting strength of comparisons. However, while these differences in temporal sampling might be expected to exacerbate any underlying differences in benthic habitat analysis showed no statistically significant differences within any type of cover across islands although there were trends for more live coral and CCA at Palmyra Atoll as compared to Mo’orea. This tendency could, however, be an artifact of temporal differences in sampling, noting that there were significant coral die off events in both regions, but particularly French Polynesia, in 2019.

**Text A2. Resighting of *A. triostegus***

*Acanthurus triostegus* resight surveys were conducted at a single site on Mo’orea, French Polynesia (17°28'47.1"S 149°47'37.1"W). A total of 12 surveys were conducted between 29-September-2019 and 22-October-2019. Any shoals and solitary fish were photographed for later analysis. Towards later surveys, individual solitary and paired fish were easily identifiable by observer and their presence was logged without photographing.

Shoaling and solitary fish in photographs were identified using right-side markings only (Fig A4). We identified and re-sighted five solitary fish and seven shoaling fish. Every resighted fish was exhibiting social behavior (shoaling or solitary) across sightings. Average number of resights was 3.5 ± 2.2, with a maximum of 8 resights and a minimum of 2. The mean time span between first and last resight was 14.7 ± 6.5 days, with a maximum of 20 days and a minimum of 1 day. We also photographed putative matches for 2 solitary and 2 shoaling fish in the same location and same behavioral mode 21.5 months later; and 2 of the same solitary fish in the same behavioral mode 36 months later. However, the shoaling fish observed at the 21.5 month observation point exhibited some subtle growths to their melanistic patterns, as such without knowledge on how these patterns may change over time, we cannot provide complete certainty that these are the same fish (Fig A4). Future work can help further substantiate if this behavioral fidelity does indeed persist for long time periods and whether this pattern remains consistent across a wider range of geographic sites.

**Figure A4.** Examples of the distinguishing markings of A. triostegus found on the reefs of Mo’orea. Top image shows a fish with distinct melanistic facial markings and bottom image shows a fish with distinguishing spots on the right flank, distinct spotting along the third bar, and an incomplete bar in the caudal peduncle.

**Figure A5.** A and B) Two shoaling *A. triostegus* sighted in 2019 (first column) and their potential resights in 2021 with growth to melanistic patterns. Fish A shows a fish with a dark mottling that contains a light vacuoule after the second bar, a thick third bar, and a notch on the ventral side of the caudal fin in 2019. In 2021 the caudal fin notch is still present, the third bar appears a bit thicker, and the mottling behind the second bar has grown, but the vacuole is still present. Fish B in 2019 has dark patterns along the nose and in the forehead area between the first and second bar, thin bars along the body, and then a thick stripe of mottling between the 5th and 6th bars, extending out into the caudal peduncle. The dorsal side of the caudal fin also has dark mottling. In 2021 the facial markings appear to have grown to almost fully occupy the anterior side of the second bar. The stripe along the posterior end appears similar, although the caudal fin has been damaged and it is not possible to assess the markings along its dorsal side. C) A solitary *A. triostegus* sighted in 2019 and potential resight in 2022 with same melanistic patterns. The fish shows an interruption along the dorsal portion of the 5^th^, an incomplete bar in at the caudal peduncle, and otherwise complete bars with no mottling.
